# Location, Location, Location: Establishing Design Principles for New Antibacterials from Ferric Siderophore Transport Systems

**DOI:** 10.3390/molecules29163889

**Published:** 2024-08-16

**Authors:** Vivien Canran Luo, Mark W. Peczuh

**Affiliations:** Department of Chemistry, University of Connecticut, 55 N. Eagleville Road, U3060, Storrs, CT 06269, USA; canran.luo@uconn.edu

**Keywords:** iron transport, siderophore, Trojan Horse, antibiotics, cefiderocol

## Abstract

This review strives to assemble a set of molecular design principles that enables the delivery of antibiotic warheads to Gram-negative bacterial targets (ESKAPE pathogens) using iron-chelating siderophores, known as the Trojan Horse strategy for antibiotic development. Principles are derived along two main lines. First, archetypical siderophores and their conjugates are used as case studies for native iron transport. They enable the consideration of the correspondence of iron transport and antibacterial target location. The second line of study charts the rationale behind the clinical antibiotic cefiderocol. It illustrates the potential versatility for the design of new Trojan Horse-based antibiotics. Themes such as matching the warhead to a location where the siderophore delivers its cargo (i.e., periplasm vs. cytoplasm), whether or not a cleavable linker is required, and the relevance of cheaters to the effectiveness and selectivity of new conjugates will be explored. The effort to articulate rules has identified gaps in the current understanding of iron transport pathways and suggests directions for new investigations.

## 1. Antibiotic Development and Microbial Iron Assimilation

Antibiotic resistance has been a long-standing hurdle in developing treatments for bacterial infections. In the United States, 2.8 million antibiotic-resistant infections resulting in approximately 36,000 deaths were estimated by the U.S. Centers for Disease Control and Prevention (CDC) in 2019 [1]. Multidrug-resistant (MDR) bacteria are notorious for their resistance to at least three classes of antibiotics such as β-lactams, oxazolidinones, and fluoroquinolones [2]. Recently, the World Health Organization (WHO) released the 2024 Bacterial Priority Pathogen List containing MDR bacteria for which new antibiotics are urgently needed [3]. The list contains the ESKAPE pathogens—*Enterococcus faecium*, *Staphylococcus aureus*, *Klebsiella pneumoniae*, *Acinetobacter baumannii*, *Pseudomonas aeruginosa*, and *Enterobacter* spp. [4]. ESKAPE pathogens are known for their ability to ‘escape’ antibiotic action [5]. They are the most common causative pathogens for nosocomial infections with symptoms such as urinary tract infection (UTI), pneumonia, meningitis, bloodstream infections, and lung infections in patients with cystic fibrosis [6]. The resistance mechanisms of the ESKAPE pathogens include drug inactivation by alteration [7,8,9], modification of drug binding sites [8,10,11], loss of porins [12,13], overexpression of efflux pumps [14,15,16,17], and biofilm formation [18,19]. In addition, the ESKAPE pathogens are able to transfer their antibiotic resistance genes to other cells, exerting even more antibacterial pressure [20]. Despite the increasing numbers of new antibiotics developed and used clinically, alternative approaches to circumvent antibiotic resistance are still urgently needed. In this review, we revisit the Trojan Horse strategy of antibacterial development and attempt to refine it by suggesting location as a design consideration. We emphasize that, when the siderophore and the warhead of a given conjugate are matched in terms of localization and target of action, more effective antibiotics arise. To accomplish this, we introduce siderophores and the protein machinery for transporting iron from outside to inside a Gram-negative bacterial cell. A number of siderophore–warhead conjugates are then surveyed, finishing with an analysis of cifederocol, a Trojan Horse antibiotic recently approved for use in the clinic to treat troubling Gram-negative bacterial infections.

Among the six aforementioned ESKAPE pathogens, the latter four are Gram-negative bacteria. In comparison to Gram-positive bacteria, Gram-negative bacteria employ an additional outer membrane that provides an extra layer of defense against antibiotics. In an effort to overcome antibacterial resistance, the ESKAPE pathogens have been subjected to extensive studies. Numerous virulence factors that facilitate colonization in hosts and subsequent mechanisms of pathogenesis have been identified [21]. Among the various virulence factors, iron acquisition is one that has gained widespread interest. Iron is an essential nutrient for all organisms because it takes part in a variety of biological processes, including cellular respiration, metabolism, and DNA repair [22]. Despite being one of the most abundant elements on Earth, the available iron required to sustain biological systems is scarce. Under aerobic conditions, iron exists as ferric ion Fe(III) with extremely low aqueous solubility (K_sp_ = 10^−18^ M at pH 7.0) [23]. To acquire extracellular iron, bacteria synthesize and secrete low-molecular-weight iron chelators called siderophores [24]. Siderophores show high affinity towards Fe^3+^ and form soluble, octahedral ferric complexes. Upon complexation, the Fe(III)–siderophore complex is available for microbial uptake [25].

**Figure 1:**
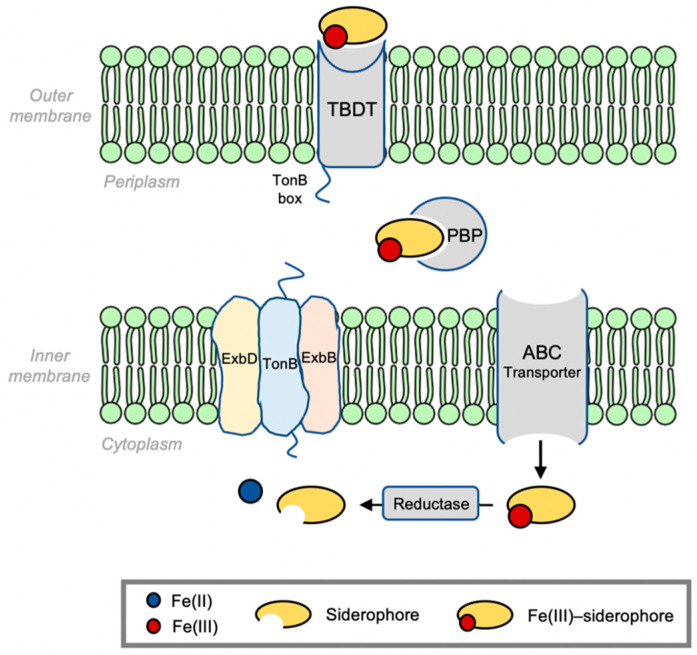
Generalized process of siderophore-mediated microbial iron uptake in Gram-negative bacteria.

To support their growth and replication, Gram-negative bacteria release siderophores that facilitate uptake of iron from the environment. The general process is summarized in Figure 1. It depicts the internalization of an Fe(III)–siderophore complex from the extracellular milieu inward to the cytoplasm. First, the complex is recognized by an outer membrane receptor specific for the given Fe(III)–siderophore complex [26]. These TonB-dependent transporters (TBDTs) share a similar architecture that includes a 22-stranded β-barrel domain, extracellular loops, an *N*-terminal plug domain inside the barrel interior, dividing it into an extracellular pocket and a periplasmic pocket, and a conserved five-residue loop termed the TonB box. When the complex binds, the TBDT transduces a signal across the membrane that leads to the unfolding of the TonB box that then engages the C-terminal of TonB in the inner membrane protein complex TonB-ExbB-ExbD [27,28]. In this way, the TBDT acquires energy in the form of proton motive force to actively transport its substrate into the periplasm [26,29]. Once through the outer membrane, a periplasmic binding protein (PBP) chaperones the complex toward the cell membrane where it complexes with an ATP-binding cassette (ABC) transporter to actively deliver the Fe(III)–siderophore complex into the cytoplasm [30]. ABC transporters shuttle a variety of substrates such as antibiotics, vitamins, and ions in addition to Fe(III)–siderophores across membranes [31]. A typical ABC transporter consists of two transmembrane domains (TMDs) that form a central translocation channel and two nucleotide-binding domains (NBDs) in the cytoplasm where ATP hydrolysis takes place to provide energy for active transport [32,33]. Once in the cytoplasm, when Fe(III) undergoes reduction, the siderophore dissociates away and Fe(II) is taken up by cytoplasmic proteins [34].

**Figure 2:**
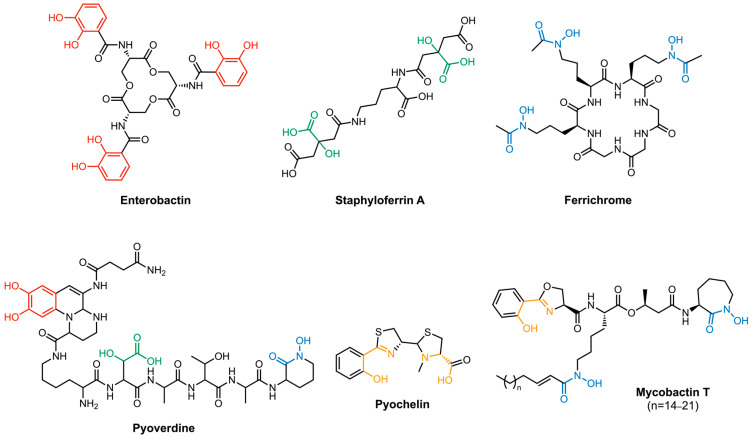
Examples of siderophores containing various iron-chelating units (red: catecholate, green: α-hydroxycarboxylate, blue: hydroxamate, and orange: other iron-chelating moieties).

Siderophores themselves are constructed primarily from metabolites that have been repurposed to coordinate iron. They use structural motifs such as catecholates, α-hydroxycarboxylates, and hydroxamates to chelate iron. A given siderophore may use only one motif to coordinate iron in an octahedral manner or it may mix and match chelating motifs. For example, enterobactin, staphyloferrin A, and ferrichrome (Figure 2) exclusively contain catecholates, α-hydroxycarboxylates, and hydroxamates, respectively. On the other hand, mixed siderophores such as pyoverdine, pyochelin, and mycobactins contain a mixture of iron-chelating units that bind iron via other *N*- and *O*-containing moieties [35]. Each Fe(III)–siderophore complex possesses unique structural and electronic features (i.e., the charge associated with the complex) that give rise to the affinity and specificity of the corresponding transport protein. In most siderophore transport systems, iron reduction takes place once the Fe(III)–siderophore complex enters the cytoplasm. This process is mediated by a cytoplasmic reductase, resulting in the release of Fe(II) [34]. This mechanism of iron release is seen in the transport of acinetobactin in *A. baumannii* [36], and ferrichrome [37] and enterobactin in *E. coli* [38]. On the other hand, the pyoverdine-mediated reduction and subsequent release of Fe(II) takes place in the periplasmic space of *P. aeruginosa* where Fe(III) is reduced by an inner membrane reductase. The Fe(II) that is released is delivered to the inner membrane receptor by a PBP [39,40]. The differences in siderophore recognition and the mechanism of iron delivery highlight the needs for targeted strategies. Consideration of the details of these processes is key to understanding the rationale behind the Trojan Horse strategies that can target a specific bacterial species.

Some bacteria do not biosynthesize siderophores themselves but nonetheless have need for iron for metabolism. They consequently express membrane receptors that recognize structural features of siderophores produced by other microbes (xenosiderophores) so they can assimilate iron [41]. Others express specific membrane receptors for both endogenous siderophores and xenosiderophores to maximize their chance of iron uptake. In response to this practice of iron thievery, some microbes produce sideromycins where a bactericidal unit is covalently linked to a siderophore. Sideromycins exploit the siderophore transport systems to enter the cell to release the antibiotic and consequently inhibit their target. This counterattack tactic helps the sideromycin-producing microbe eliminate competition for iron. Such an approach where a sideromycin utilizes the siderophore transport system to inhibit its intracellular target is known as the Trojan Horse approach. For that reason, sideromycins, or siderophore–drug conjugates, are often referred to as Trojan Horse antibiotics.

**Figure 3:**
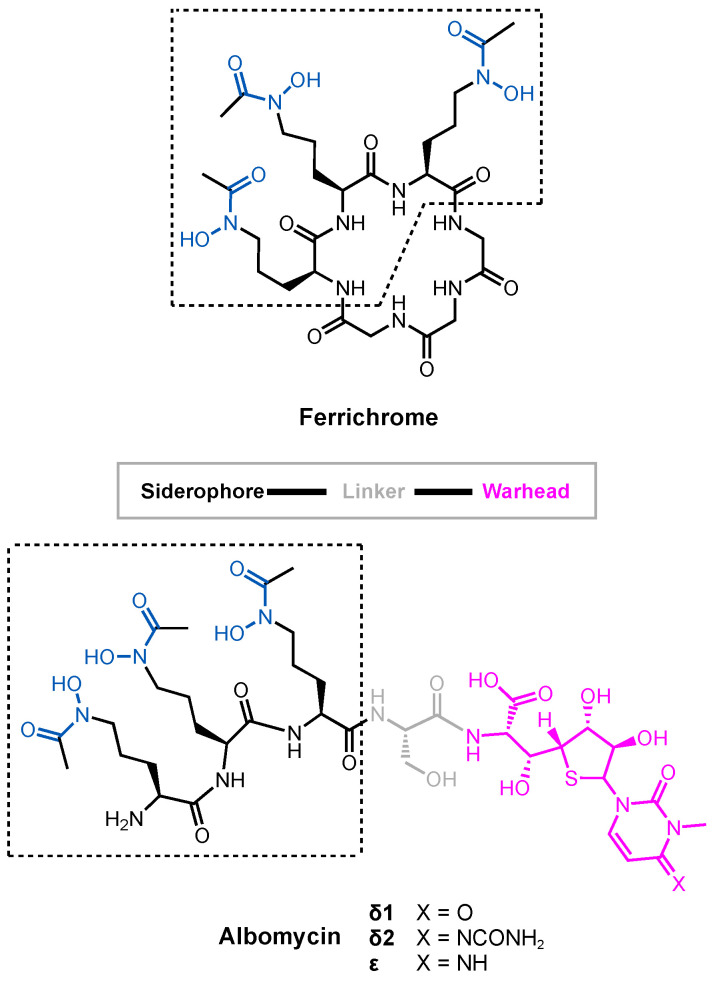
Structural comparison of ferrichrome and albomycins (blue: hydroxamate, grey: linker, pink: warhead).

Albomycin (Figure 3) is a prototypical Trojan Horse antibiotic created by nature. It was originally isolated from *Streptomyces griseus* in 1947 and remains one of the most studied sideromycins due to its early discovery [42]. Albomycin exists as a mixture of structurally related compounds, albomycin δ1, albomycin δ2, and albomycin ε [43]. Functionally, these compounds combine a warhead (a potent thionucleoside tRNA synthase inhibitor) and a siderophore analog (tris-hydroxamate) through a linker unit (D-serine) in one molecule [44]. Albomycin uses the ferrichrome uptake system in *E. coli* to enter the cell. Once it enters the cytoplasm, a peptidase releases the antibacterial warhead to engage its target [45]. Due to its unique method of cell entry, albomycin shows an inhibitory effect ten times stronger than that of penicillin against *E. coli* [46].

There has been a sustained effort to assemble a library of designed, synthetic sideromycins as an alternative clinical solution to antibacterial resistance. To understand the molecular design principles that allow them to deliver antibiotic warheads to the right target in Gram-negative pathogens, we ought to first understand different siderophore uptake systems. What follows is a number of representative siderophores with various iron-chelating units that utilize distinct siderophore transport systems in *A. baumannii*, *E. coli*, and *P. aeruginosa*. *A. baumannii* and *P. aeruginosa* are both ESKAPE pathogens that have been the subject of studies where a variety of siderophores have been identified. As the most well-studied model organism, *E. coli* expresses multiple siderophore transport systems that are characterized in detail. In the meantime, the Gram-negative ESKAPE pathogen *K. pneumoniae* and *E. coli* share a similar profile of endogenous siderophores and uptake systems [47]. Understanding iron acquisition in *E. coli* will in turn help us understand similar processes in *K. pneumoniae*. Based on publications detailing the studies of each siderophore transport pathway, we aim to assess not only the sequence of events during uptake but also the physical interactions that give rise to the affinity and selectivity of siderophores for their corresponding transport proteins. Specifically, we will explore the following siderophore uptake systems: (i) *A. baumannii* pre/acinetobactin transport where two structurally related isomers are responsible for assimilating iron under different environmental conditions; (ii) the *E. coli* ferric hydroxamate uptake that transports xenosiderophores such as ferrichrome but is also the main target for albomycin; (iii) the enterobactin uptake systems in *E. coli* and *P. aeruginosa* where the two pathogens utilize the same siderophore but express independent pathways with distinct affinities; and (iv) the transport of two mixed-type siderophores, pyoverdine and pyochelin, in *P. aeruginosa.* The siderophores involved in these pathways exhibit a wide range of structural variations. Studying these siderophores and their transport will facilitate the construction of creative guidelines for designing novel sideromycins to introduce antibiotic warheads into the cell to reach the right target.

## 2. Bacterial Infrastructure of Iron Siderophore Transport

**Figure 4:**
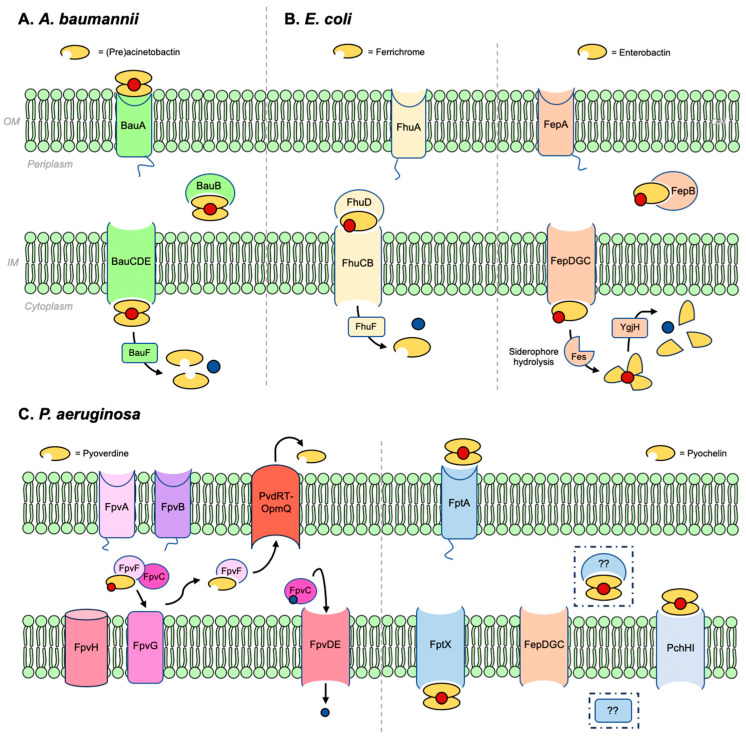
Selected siderophore uptake systems in (**A**) *A. baumannii*, (**B**) *E. coli*, and (**C**) *P. aeruginosa*.

Specifics in terms of the protein machinery involved in siderophore-mediated iron transport into Gram-negative bacteria are presented here. Similar to the general process shown in Figure 1, details of iron transport mediated by (pre)acinetobactin in *A. baumanii*, ferrichrome, and enterobactin in *E. coli*, and pyoverdine and pyochelin in *P. aeruginosa* are shown in Figure 4. Organizing the names and functions of these proteins (Table 1) is relevant to the explanation of the action of sideromycin-inspired Trojan Horse antibiotics that follows.

### 2.1. Acinetobacter baumannii

*Acinetobacter baumannii* (*A. baumannii)* targets the skin and mucous membranes across a wide range of anatomical regions, including the upper respiratory tract [48,49,50,51]. It has been categorized as a ‘red alert’ human pathogen due to its high antibiotic resistance [52]. Clinical symptoms associated with *A. baumannii* infections include pneumonia [53,54], bloodstream infections [55], and meningitis [56]. Among structurally distinct siderophores produced by *A. baumannii*, the pH-dependent isomeric pair preacinetobactin and acinetobactin has been tagged as a virulence factor [57,58].

#### 2.1.1. Preacinetobactin and Acinetobactin

**Figure 5:**
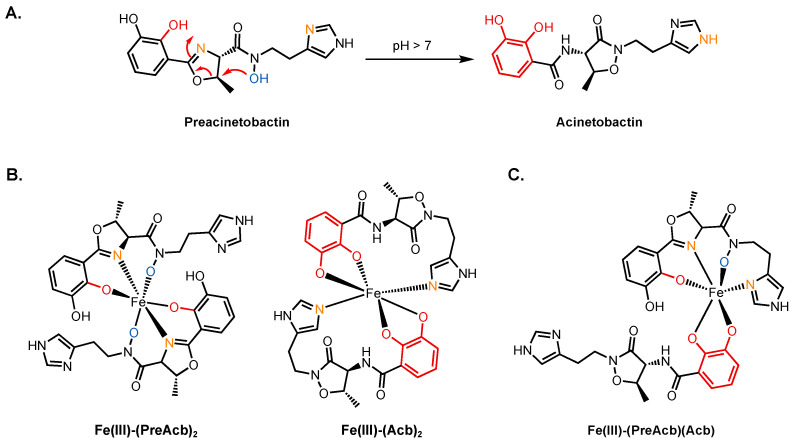
(**A**) Isomerization of preacinetobactin to acinetobactin. (**B**) Fe(III)–(PreAcb)_2_ and Fe(III)–(Acb)_2_ complexes. (**C**) Mixed Fe(III)–(PreAcb)(Acb) complex.

Preacinetobactin (PreAcb, Figure 5) contains catecholate, oxazoline, hydroxamate, and imidazole moieties that can complex iron. It binds in a 2:1 stoichiometry with Fe(III) to give a complex with an overall charge of −1 and an affinity constant of *K*_f_ = 10^27.4^ M^−2^. Its isomer, acinetobactin (Acb), contains just the catecholate and imidazole units for coordination. Acb too makes a 2:1 complex with a −1 charge and an affinity constant of *K*_f_ = 10^26.2^ M^−2^. PreAcb is the product of biosynthesis and persists at pH values less than 7. A rapid, irreversible, non-enzymatic isomerization occurs above neutral pH to produce Acb (Figure 5A) [59]. Different strains of *A. baumannii* grow over a range of pH (5–8) so both PreAcb and Acb are assumed to play a role in iron acquisition. *A. baumannii* infection sites are frequently acidic (pH~5), making PreAcb the most likely siderophore operative here [60,61]. (Pre)acinetobactin binds Fe(III) with a 2:1 stoichiometry, and both are capable of supplying the cell with iron [59,62]. Density function theory (DFT)-calculated structures of the two Fe(III)–siderophore complexes Fe(III)–(PreAcb)_2_ and Fe(III)–(Acb)_2_ showed that the ligands coordinate to the iron center through four oxygen atoms and two nitrogen atoms, giving rise to a negative net charge in the complex (Figure 5B) [63]. The Fe(III)–(PreAcb)_2_ complex is stable at pH < 7.5 and no isomerization was observed because the hydroxamate hydroxy group took part in iron chelation, which is unable to initiate isomerization. On the other hand, the Fe(III)–(Acb)_2_ complex was unstable under acidic conditions as the catecholate ligand becomes protonated, reducing its ability to bind iron [59]. As a result, Acb is mostly responsible for assimilating iron under neutral or basic conditions.

In the (Pre)Acb pathway (Figure 4), only one outer membrane TBDT (BauA) has been identified and it is assumed that both siderophores utilize this transporter. Nevertheless, experimental results obtained by two separate teams showed ambiguity regarding whether both Fe(III)–(PreAcb)_2_ and Fe(III)–(Acb)_2_ utilize the same TBDT [59,62,64]. Wencewicz and co-workers reported that a ∆*bauA* mutant failed to use the two siderophores as sources of iron [59]. Others, however, observed a loss of Fe(III)–(PreAcb)_2_ uptake in ∆*bauA*, yet the mutant could grow efficiently when treated with Fe(III)–(Acb)_2_ [62]. A crystal structure of BauA showed that it preferentially binds the 1:1:1 complex Fe(III)–(PreAcb)(Acb) (Figure 5C). The binding pocket of BauA is hydrophobic and the fact that PreAcb and Acb form Fe(III)–siderophore complexes with the same (–1) charge suggests that physical compatibility might play a significant role in substrate recognition. BauA interacts predominantly with PreAcb of the hetero-complex, forming hydrogen bonds to its hydroxamate group using Tyr312 and Arg253 [65]. A molecular model suggested that Acb would not fit into the binding pocket without significant clash with Tyr312. Under physiological conditions, the hetero-complex Fe(III)–(PreAcb)(Acb) is expected to predominate in areas such as the bloodstream where free iron is scarce. At acidic *A. baumannii* infection sites, Fe(III)–(PreAcb)_2_ is expected to be dominant. The crystal structure partially detangled the mystery regarding the transport of Acb. Perhaps, in the presence of both PreAcb and Acb, BauA transports the 1:1:1 complex. Fe(III)–(Acb)_2_ may not be recognized by BauA but may use another, as-of-yet undiscovered outer membrane receptor to supply iron.

Once the complex arrives in the periplasm, it is chaperoned by the PBP BauB to the inner membrane complex BauCDE [66]. The complex is translocated into the cytoplasm by BauCDE, followed by reduction and liberation of iron by the reductase BauF [36]. A crystal structure of BauB bound to Fe(III)–(Acb)_2_ revealed that the protein had the characteristic structure of a PBP with two globular α/β domains connected by an α-helix. The complex binding site was situated between the two α/β domains [67]. The coordination of Acb to iron in Fe(III)–(Acb)_2_ is consistent with that calculated by DFT [63]. One Acb molecule in the complex was exposed to solvent, while the other one was found inside the binding pocket with the catecholate ring partially exposed. Mediated by water molecules, the carbonyl group attached to the exposed catecholate ring of the buried Acb interacted with Asp83, Ile104, and Val105 at the binding site. The methyl group from the heterocycle was found in a pocket defined by side chains of Tyr84, Val264, and Tyr301. The interactions between the buried Acb and the binding pockets leave little room for any modification to attach an additional antibiotic warhead on the buried molecule. On the other hand, the exposed Acb molecule only made two polar interactions with the protein: the isoxazolidinone carbonyl oxygen coordinated to Arg217 through a water molecule and the C3 catecholate oxygen, despite coordinating iron, was also involved in a hydrogen bond with Tyr301. The position of the exposed Acb molecule offers an opportunity for functionalization. It is important to acknowledge, though, that in an Fe(III)–siderophore complex, only one of the two siderophore molecules can be modified with a warhead to ensure substrate recognition. In other words, the 2:1 complex would possess two distinct ligands, an unmodified Acb and another with a warhead. Interestingly, BauB transports not only PreAcb and Acb complexes but also fimsbactin A (Fim, vide infra), another siderophore used by *A. baumannii*. BauB displays lower selectivity in terms of ligands it binds in comparison to its TBDT partner BauA [66,67]. To be able to utilize the same PBP, the substrates must share some molecular similarities.

The crystal structures of BauA and BauB offered insights into where PreAcb and Acb can be modified into appropriate siderophore–drug conjugates. As the PreAcb hydroxamate group is directly involved in recognition with BauA, the imidazole ring and the catechol ring are reasonable candidates to be functionalized without interfering with substrate recognition. Song carried out a study of PreAcb analogs to test their abilities to bind iron and mediate iron uptake [62]. Consistent with the BauB crystal structure, the C3 position of the catecholate ring and the alkylation of either of the two imidazole nitrogen atoms did not prevent iron chelation or growth promotion. Removal of the 3-hydroxyl group on the catecholate ring did not perturb the function of the siderophore. However, methylation of the hydroxamate oxygen did not interfere with iron chelation but failed to promote cell growth. This is consistent with the fact that the hydroxamate group is key in substrate recognition and any structural modification would prevent the uptake of the siderophore. Analogs of Acb examined by Wencewicz demonstrated that the 2,3-dihydroxylcatechol motif was indispensable while the imidazole group was not required for Fe(III) binding [68]. We reported the synthesis of 5-phenyl PreAcb and demonstrated its ability to bind iron [69]. Nevertheless, the resulting ferric complex was not able to promote the growth of *A. baumannii* 19601-s1, a *basD* mutant that lacks the biosynthesis gene for producing PreAcb. The failed growth recovery by 5-phenyl PreAcb could be ascribed to the lack of, or severely obstructed binding to, BauA or BauB. It was also possible that the poor solubility of the ferric complex in water limited the availability of the complex to the cell.

#### 2.1.2. Fimsbactins

**Figure 6:**
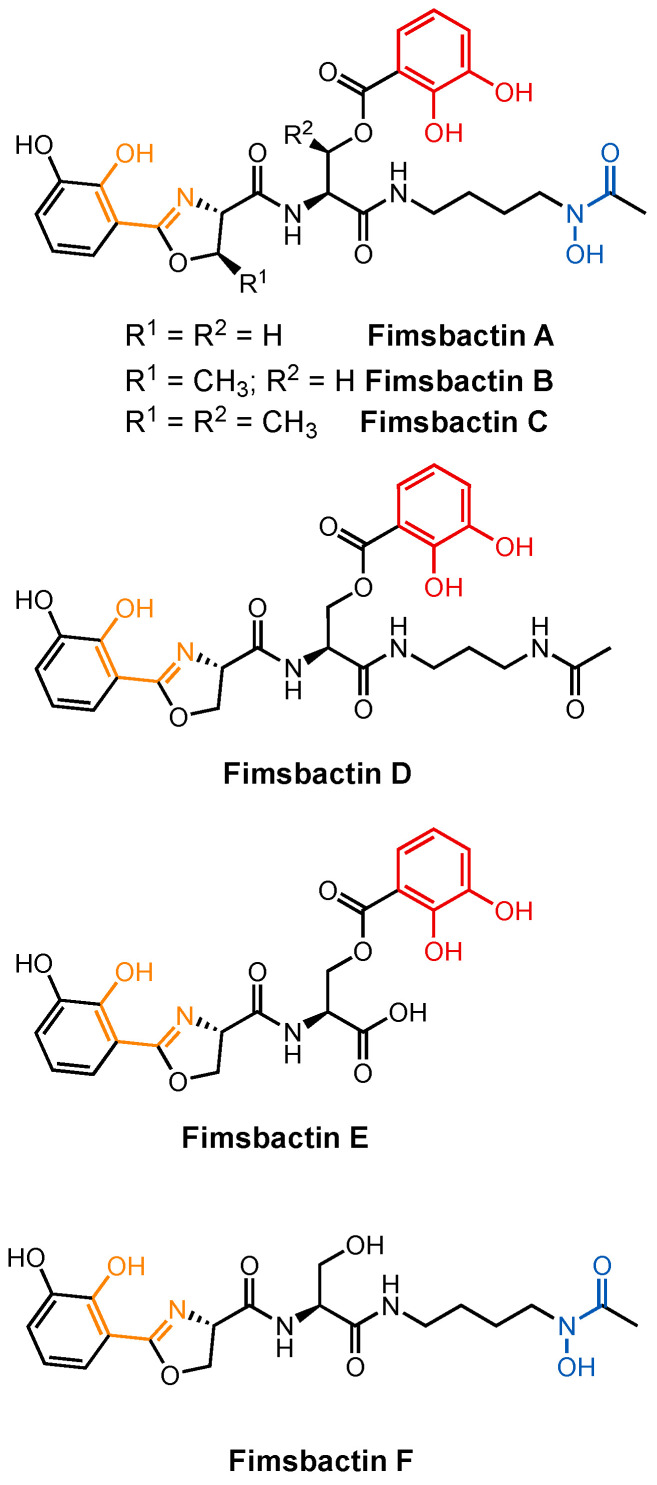
Structures of fimsbactin A–F (red: catecholate, blue: hydroxamate, and orange: other iron-chelating moieties).

Fimsbactin A (Fim) was among a mixture of structurally similar fimsbactins isolated from the pathogenic clinical isolate *A. baumannii* ATCC 17978 (Figure 6) [70]. Reminiscent of preacinetobactin, Fim coordinates iron through its phenolate–oxazoline (using only one of two catecholate oxygens), catecholate, and hydroxamate units. It forms a 1:1 complex with Fe(III) with an overall charge of −1. At *K*_f_ = 10^27.1^ M^−1^, the affinity of Fim towards Fe(III) is similar to that of PreAcb. Although the fimsbactin biosynthetic gene cluster was also identified in *A. baumannii* 6013150, no fimsbactins have been isolated from any other strains of *A. baumannii*, indicating that fimsbactins are not essential for virulence. Although Fim has been shown to be able to promote the growth of *A. baumannii* under iron-deficient conditions, a dedicated Fim transport pathway has yet to be identified. A series of DFT calculations using the BauB crystal structure revealed that the common phenolate–oxazoline and catecholate units in Fim and (Pre)Acb were well aligned. This, along with the fact that Fe(III)–Fim is bound by BauB in competition with Fe(III)–((Pre)Acb)_2_ complexes, suggests other parts of the same pathway may be operative [63]. However, the decision points and process that regulate which siderophore is utilized under a given set of conditions remains a lingering question for *A. baumannii* and in the field broadly.

### 2.2. Escherichia coli

*E. coli* is a Gram-negative, rod-shaped bacterium commonly found in the lower intestine of warm-blooded species [71,72]. Most *E. coli* strains are commensal, but some can cause severe gastrointestinal diseases [73]. *E. coli* is also one of the most common causative pathogens for urinary tract infections (UTIs). In some cases, UTI patients exhibit conditions such as bacteremia, septicemia, and urosepsis that sometimes result in death [74,75].

**Figure 7:**
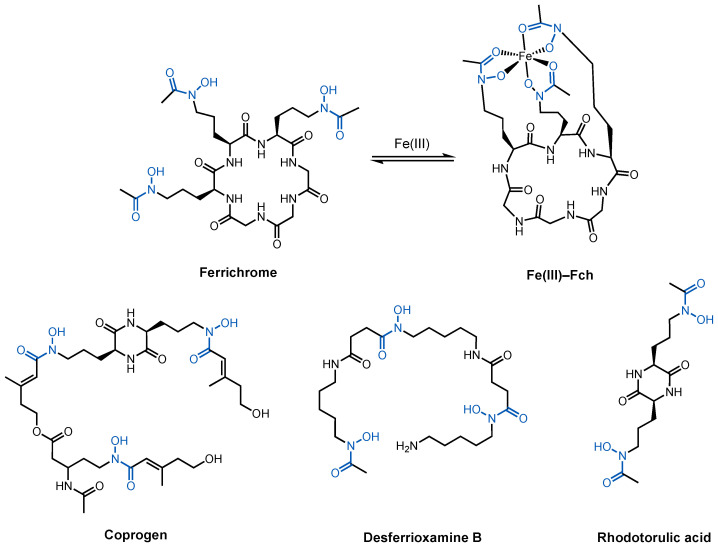
Hydroxamate (blue) siderophores that utilize the Fhu pathway in *E. coli*.

As a model organism, the processes that take place in *E. coli*, including iron uptake, have been extensively studied. *E. coli* utilizes a wide range of endogenous and exogenous siderophores. Commensal strains of *E. coli* may only produce enterobactin [76]. Pathogenic strains can also produce salmochelin, yersiniabactin, and aerobactin to maximize iron uptake under iron-deficient conditions [77,78,79]. Moreover, a given strain may also express siderophore transport systems for xenosiderophores such as ferrichrome and coprogens (Figure 7) [80,81]. This section focuses on (i) ferrichrome and coprogen uptake via the ferric hydroxamate uptake (Fhu) pathway, which is also the main entry route for albomycin, and (ii) enterobactin, the tris-catecholate siderophore that is produced and utilized by all strains of *E. coli*. The siderophores involved in these two pathways bear distinct structural features and electronic properties. Understanding these pathways can help us understand the diversity of siderophores and different possible approaches to developing siderophore–antibiotic conjugates targeting *E. coli*.

#### 2.2.1. Ferrichrome and Related Hydroxamates

Hydroxamate siderophores are produced by fungi and some strains of bacteria but not *E. coli* [82,83,84]. *E. coli* does, however, express Fhu proteins for hydroxamate uptake as xenosiderophores such as ferrichrome and the coprogens, a family of linear hydroxamate siderophores, to acquire iron (Figure 7). Utilizing xenosiderophores greatly alleviates the biosynthetic burden on bacteria to rely on endogenous siderophores to assimilate iron. Ferrichrome (Fch) is a tris-hydroxamate siderophore with an affinity constant of 10^29^ M^−1^ for Fe(III) [85,86]. With its three hydroxamates, it forms a neutral, 1:1 complex with Fe(III) at physiological pH (Figure 7). As a cyclic peptide, Fch contains both polar and hydrophobic character associated with its structure. Similarly, linear tris-hydroxamate siderophores coprogen and desferrioxamine B also form neutral 1:1 complexes with Fe(III), whereas rhodotorulic acid forms a 3:2 complex to fulfil the octahedral coordination sphere around Fe(III) [87]. These hydroxamate siderophores also utilize the Fhu pathway to enter the cytoplasm of *E. coli*. The Fhu pathway involves two TBDT receptors, FhuA and FhuE. FhuA is responsible for transporting Fch and albomycin [88,89,90,91,92], whereas FhuE transports the coprogens. In the periplasm, the PBP FhuD chaperones the Fe(III)–siderophore complexes to FhuCB, the inner membrane permease. Finally, Fe(III) is reduced to Fe(II) in the cytoplasm by FhuF and released to intracellular proteins.

FhuA is the outer membrane TBDT for Fch and is also exploited by the albomycins. Like other TBDTs, FhuA is a 22-stranded transmembrane β-barrel protein with a plug domain inside the inner passage. The plug is connected to the β-barrel through a network of hydrogen bonds and undergoes conformational changes that enable the passage of small-molecule substrates in an energy-dependent manner. A substrate binding site near the external pocket is lined with complementary residues, including Arg81, Gln100, and Tyr116, that can tightly associate with Fe(III)–Fch. These residues are highly conserved among Fch receptors from *E. coli*, *Pantoea agglomerans*, *Salmonella paratyphi* strain B, and *Salmonella typhimurium* [93]. The ligand binding site has a high density of aromatic residues. The δ+ end of the dipoles of Fch’s amides, localized by its ring, make favorable interactions with the π-cloud of the aromatic residues. Since the Fe(III)–Fch complex is neutral, interacting with the FhuA binding site that is in line with neutral, aromatic residues is favorable. A negatively charged complex, such as Fe(III)–Ent, presented in the next section, would be less favorable [89].

The other TBDT, FhuE, selectively recognizes coprogens [81], a family of linear hydroxamate siderophores, including coprogen, desferrioxamine B (DFB), and rhodotorulic acid (Figure 7) [94]. FhuE imports coprogen with the highest affinity, followed by rhodotorulic acid and DFB [95]. FhuE is unable to recognize cyclic hydroxamate siderophores such as Fch [81,96]. Coprogen and DFB both contain three bidentate hydroxamate units, giving rise to 1:1 ferric complexes. Rhodotorulic acid contains only two hydroxamate units so a 3:2 (ligand-to-metal) stoichiometry is preferred. The binding modes of FhuE with the complexes were simulated in silico. Ferrioximine B and Fe(III)–coprogren took up similar poses in the study as well as one rhodotorulic acid in its complex. The lower affinity of FhuE towards ferrioxamine B and Fe(III)–rhodotorulic acid was ascribed to the lower physical compatibility within the binding pocket. For the same reason, the binding pocket in FhuE could not accommodate Fe(III)–Fch without clashes. The key coordination between Arg142 in the binding site and the hydroxamate group is only possible when the siderophore is planar. Fe(III)–Fch would not fit into the binding pocket without clashing with Arg142 [95]. Expression of FhuA and FhuE by one organism suggests an opportunistic approach the pathogen takes to maximize its chances of iron intake. For example, when albomycins targets FhuA that will inevitably suppress Fch uptake, FhuE can still function as an alternative route to supply the cell with iron.

In the periplasm, FhuD is the PBP that escorts complexes to the inner membrane ABC transporter FhuCB. A crystal structure of FhuD revealed a kidney-bean-shaped protein, consistent with the typical structure of a Class III PBP [30]. FhuD is able to transport both linear and non-linear hydroxamate Fe(III)–siderophore complexes, including Fch, DFB, and coprogen [97,98]. Substrate recognition was mediated by hydrophilic and hydrophobic interactions. A series of hydrophobic residues such as Trp, Tyr, Ile, and Leu on one side of the binding pocket interacts with the aliphatic carbon atoms in hydroxamate siderophores. The side chain of Arg84 formed hydrogen bonds with two of the three carbonyl oxygens of the hydroxamate units, whereas the other carbonyl oxygen formed a hydrogen bond with Tyr106. A similar hydrogen bonding network involving Arg and Tyr is observed in FhuA. In comparison, FhuA and FhuE possess an extensive network of hydrogen bonds with higher degrees of selectivity towards siderophores.

The inner membrane transporter FhuCB is a type II ABC importer [99]. As FhuD complexes with FhuCB, the substrate binding pocket in FhuD is infiltrated by two loops from the transmembrane domain FhuB to confiscate its Fe(III)–siderophore cargo. The interactions between FhuD and FhuB involve specific residues and an extensive hydrogen bonding network. Based on calculations [100], the central pathway opens inwards, facing the cytoplasm. The residues in the central pathway are categorized into three types: hydrophobic residues with small side chains, polar residues that are able to form hydrogen bonds, and methionine (Met) residues. Fch forms favorable interactions with aromatic residues that are absent in the FhuB inner channel [89]. This indicates weak interactions with the substrate and therefore rapid translocation. The relatively high abundance of polar residues helps the substrate to exit the pathway. The roles of Met residues are unclear, but mutagenesis showed them to be important for transport. The sulfur atoms in Met side chains could potentially interact with Fe(III), as they have a high affinity towards electrophilic centers such as iron.

Finally, the reductive release of iron is carried out by FhuF in the cytoplasm. Unlike siderophore-interacting proteins that use flavins as redox cofactors, FhuF is a ferric siderophore reductase (FSR) that contains a [2Fe–2S] cluster as the redox center. The [2Fe–2S] cluster is situated in a neutral region between two positively charged regions. When electron transfer from the reductase to the siderophore takes place, the positively charged region can release a proton to maintain charge neutrality [37]. Once Fe(III) is reduced, release of Fe(II) is rapid [34,101,102,103].

#### 2.2.2. Enterobactin and Related Catecholates

Enterobactin (Ent) (Figure 3) is an L-serine-based tris-catecholate siderophore used by several pathogens. Bacteria such as *E. coli* and *Salmonella typhimurium* carry gene clusters for biosynthesizing Ent, whereas others such as *P. aeruginosa* express an uptake pathway for Ent as a xenosiderophore [104,105,106]. Ent is one of the strongest siderophores (*K*_f_ = 10^49^ M^−1^) [107] with six negatively charged catecholate oxygens coordinating to iron, giving the Fe(III)–Ent complex a net charge of −3.

**Figure 8:**
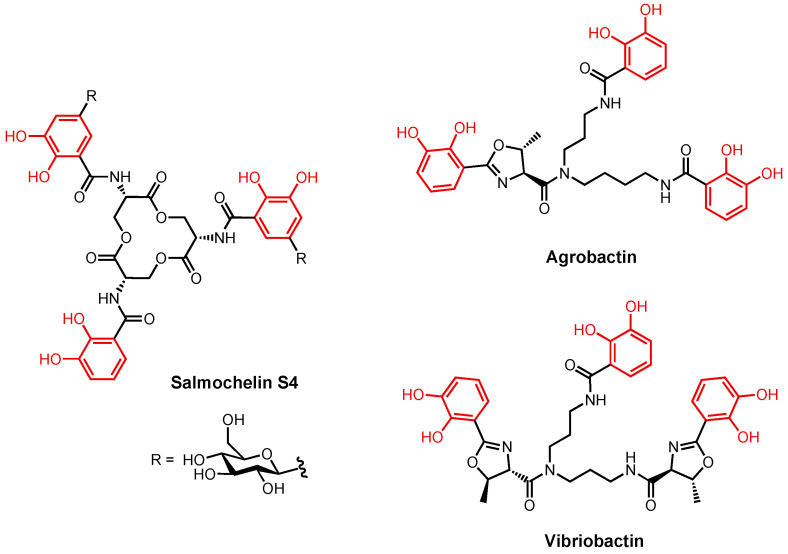
Structures of salmochelin S4, agrobactin, and vibriobactin (red: catecholate).

Ent uses the outer membrane TBDT FepA to enter the periplasm [29,108]. The binding site consists of mostly positively charged and aromatic residues [109,110]. Unlike the binding site in FhuA that contains a high density of neutral aromatic residues, the positively charged residues in FepA suggest the significance of electrostatic interactions in substrate recognition. The positively charged residues in FepA are most likely responsible for attracting the negatively charged Fe(III)–Ent complex. The aromatic residues in FepA are most likely present to facilitate the transport of the complex. In pathogenic strains of *E. coli*, Ent shares the outer membrane TBDT IroN with salmochelin, a *C*-glucosylated enterobactin (Figure 8) [111]. Other catecholate-containing siderophores use the TBDTs Fiu and CirA to cross the outer membrane [112,113]. Fiu imports a broad spectrum of catecholate siderophores [112,114,115], and the importation of compounds by Fiu is seemingly independent of iron [116,117]. Fiu is unable to transport Ent, but it was speculated that it was responsible for the re-uptake of 2,3-dihydroxybenzoyl-L-serine, the product of Ent hydrolysis [118,119]. Although the crystal structure of Fiu has been solved, the key residues in the substrate binding pocket have not been identified [120]. CirA is another versatile TBDT that transports catecholate siderophores. It also transports Colicin Ia, a 69 kDa antimicrobial protein that inhibits *E. coli* growth [121,122,123,124,125]. From a crystal structure of CirA solved with Colicin Ia as the substrate [126], the high affinity between them was ascribed to a total of 18 intermolecular hydrogen bonds. In addition, a small cluster of negatively charged residues (Asp350, Asp358, Asp362, and Glu269) in Colicin Ia were in close proximity to Arg325 and Arg490 in CirA. Although the interactions between CirA and any catecholate siderophores have not been investigated, the electrostatic interactions between the positively charged arginine residues on CirA and the negatively charged ferric catecholate siderophore complex most likely play a key role in substrate recognition.

In the periplasm, FepB is responsible for picking up Fe(III)–Ent [127] and directing it to FepDGC, the inner membrane ABC transporter [128,129]. The substrate transfer process is not yet well understood. It also remains unclear if Fiu and CirA substrates also utilize FepB to travel in the periplasm. FepDGC consists of FepC as the ATPase and FepD and FepG as the dimeric transmembrane proteins [128,129]. FepB is a Class III PBP with a similar structure to FhuD [130]. However, unlike FhuD, which accepts a variety of related cargos, FepB exclusively recognizes Ent and does not interact with the tris-catecholate siderophores agrobactin and vibriobactin (Figure 8) [131,132]. Once inside the cytoplasm and prior to the release of iron from Fe(III)–Ent, the enterobactin esterase Fes breaks down Ent into three 2,3-dihydroxybenzoyl-L-serine units [133,134]. After, it is thought that the reduction and subsequent release of iron is carried out by YgjH. YgjH is a siderophore-interacting protein that uses NADPH as its redox cofactor. Supported by mutagenesis, the YgjH substrate binding site contains indispensable basic residues (Lys55, Arg130, and Arg246) that most likely participate in electrostatic and/or cation–π interactions with the substrate [38].

### 2.3. Pseudomonas aeruginosa

*P. aeruginosa* is an opportunistic Gram-negative pathogen that threatens immunocompromised patients, including those with chronic obstructive pulmonary disease, cystic fibrosis, and cancer [135,136,137]. *P. aeruginosa* infections often develop in the blood, lungs, and urinary tract to cause diseases such as bloodstream infections, pneumonia, and UTIs [138,139,140,141]. The pathogen shows high resistance to antibiotics, including β-lactams [142,143], and it is classified as a critical pathogen by the WHO [144]. To acquire iron, *P. aeruginosa* relies mainly on pyoverdine (Pvd) and pyochelin (Pch). Pch is poorly soluble in water and therefore has a relatively low affinity for iron (*K*_f_ = 2 × 10^5^ M^−2^ in ethanol) [145]. In comparison with Pch, Pvd has significantly higher affinity for iron (*K*_f_ = 10^30.8^ M^−1^) [146]. As a result, Pvd and Pch are considered the major and minor siderophores of *P. aeruginosa*, respectively [147]. In addition, *P. aeruginosa* also utilizes Ent as a xenosiderophore [106].

#### 2.3.1. Pyoverdine and Pyochelin

Pyoverdines (Pvds) are a group of structurally related, mixed-type siderophores. They are peptides produced by non-ribosomal peptide synthetases linked to a chromophore derived from 2,3-diamino-6,7-dihydroxyquinoline that gives rise to its fluorescent properties (Figure 2) [148,149,150]. Pvds are categorized into type I, II, and III based on their peptide sequence [148]. More than 100 different Pvds are produced by *Pseudomonas* strains [151] that contain various iron-chelating moieties. Fe(III)–Pvd complexes also vary in terms of stoichiometry and net charge. Type I Pvds use FpvA [152,153] and type II and III Pvds use FpvB to enter the periplasm [152,154]. The majority of *Pseudomonas* strains utilize endogenous Pvds and are unable to transport Pvds produced by other strains, resulting in high specificity for uptake. A crystal structure of FpvA isolated from wild-type *P. aeruginosa* was identified [155]; experiments showed that it was able to transport Pvd produced by *P. aeruginosa* ATCC 13525 and 18.1 [156,157]. Its substrate binding site consisted of predominantly aromatic residues such as Tyr and Trp that were able to interact with the hydrophobic areas of Pvd. Structures of FpvA using six different pyoverdines representing types I, II, and III revealed that the chromophore, Fe(III), and hydroxamate of each Fe(III)–siderophore complex were in nearly the same location in the binding site, suggesting the rest of the peptide was responsible for recognition by the receptor [155]. The structure of FpvB, which transports type II and III Pvds, has yet to be determined. However, it is known that FpvB also transports xenosiderophores Fch and DFB with higher affinity than Pvds [158].

In the periplasm, Fe(III)–Pvd is chaperoned by a pair of periplasmic binding proteins, FpvC and FpvF. A sequence alignment suggests FpvC is a metal-binding protein and FpvF is a siderophore-binding protein. The heterodimer can therefore bind both Fe(III)–Pvd and *apo*-Pvd [159]. FpvC and FpvF together deliver Fe(III)–Pvd to the reductase FpvG embedded in the inner membrane. As Fe(III) is reduced, Fe(II) is in turn released from the complex [39,40]. The inner membrane protein FpvH is also vital in the reduction and release of Fe(III), but its role is unclear [40]. After dissociation, FpvF recycles *apo*-Pvd by carrying the molecule to the ATP-dependent efflux pump PvdRT-OpmQ, and the siderophore is released back into the extracellular space [160,161]. In the meantime, FpvC shepherds Fe(II) to the FpvDE where it can be transported into the cytoplasm [159]. The details of this mechanism remain unclear. However, in the absence of FpvG, Fe(III)–Pvd accumulates in the periplasm and Fe(III) is unable to cross the inner membrane [40]. In other words, Fe(III) reduction is a prerequisite for its release and subsequent translocation into the cytoplasm.

The high specificity of FpvA for Pvd and its uncommon mechanism of Fe(III) reduction and release make the development of Pvd–drug conjugates challenging. For one, each strain of *P. aeruginosa* produces a unique Pvd and an FpvA tailored for its Pvd. Any changes to the native Pvd structure will likely prevent FpvA recognition, resulting in a lack of uptake. In addition, the Pvd uptake pathway only takes the siderophore as far as the periplasm. Once Fe(III) is reduced and released, the siderophore is then recycled.

**Figure 9:**
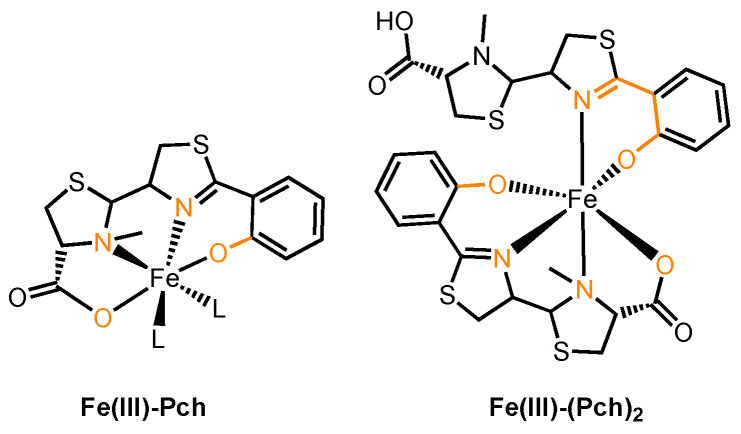
The 1:1 and 2:1 Fe(III)–Pch complexes (orange: other iron-chelating moieties).

To develop a versatile sideromycin carrying a warhead that inhibits either a periplasmic or an intracellular target, Pch is a potentially more suitable candidate. Unfortunately, its uptake is not well understood. Pch is a low-molecular-weight, mixed-type siderophore with one thiazoline and one thiozolidine that binds Fe(III) with a 2:1 stoichiometry [162]. It is hydrophobic and poorly soluble in water [163]. The crystal structure of FptA co-crystallized with Fe(III)–(Pch)_2_ showed electron density associated with only one equivalent of Pch in the substrate binding pocket (Figure 9) [164]. As four chelating atoms from one molecule of Pch were observed, an asymmetric Fe(III)–(Pch)_2_ complex where one Pch molecule contributes four chelating atoms and the other contributes only two, shown in Figure 9, was proposed. The proposed asymmetric structure of Fe(III)–(Pch)_2_ was consistent with that simulated by computational methods (Figure 9) [162]. What this in turn reveals is that only one molecule of Pch is involved in recognition and the TBDT most likely has a low selectivity for the identity of the second iron-chelating molecule. The substrate binding pocket of FptA consists of a high population of hydrophobic and aromatic residues, and this is consistent with the hydrophobic nature of Pch. In addition, residues from the plug domain, including Leu116 and Leu117, hydrogen-bond with the carbonyl oxygen atom in Pch upon substrate binding.

A PBP responsible for Fe(III)–(Pch)_2_ uptake has not been identified. By examining the complete genome of *P. aeruginosa* PAO1, it was speculated that FepB is the PBP [165]. The inner membrane permease FptX is responsible for approximately 50% of the Fe(III)–(Pch)_2_ uptake [166]. FptX appears to be a novel class of single subunit siderophore transporters that differ from ABC receptors such as FhuBC [166,167]. In the meantime, FepDGC, as well as PchHI, a heterodimeric inner membrane ABC transporter, also takes part in the translocation of Fe(III)–(Pch)_2_ into the cytoplasm [168,169]. Unlike Pvd, which only reaches the periplasm, Pch carries iron into the cytoplasm. Any antibiotics with a cytoplasmic target have the potential to be carried into the cell by a Pch–drug conjugate.

#### 2.3.2. Enterobactin

**Figure 10:**
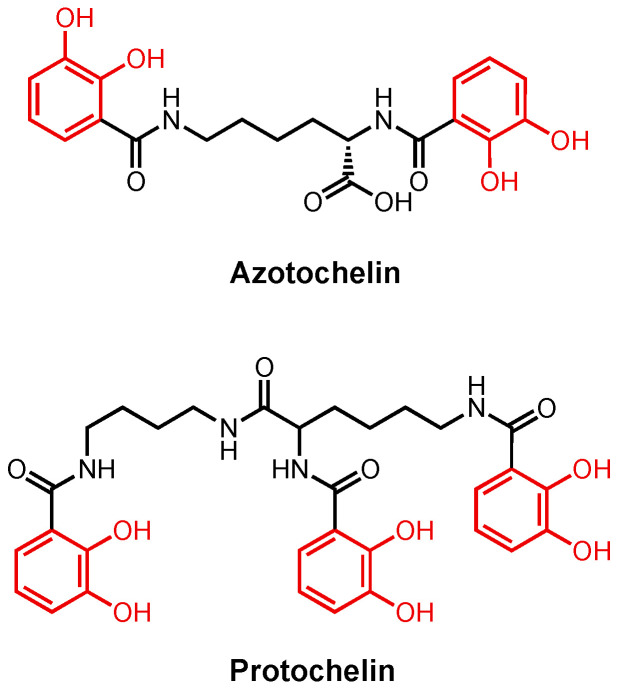
Structures of azotochelin and protochelin catecholate siderophores produced by *A. vinelandii* (red: catecholate).

In addition to Pvd and Pch, *Pseudomonas* also uses Ent as a xenosiderophore. The pathogen expresses two outer membrane TBDTs, PfeA [106,170] and PirA [171], where the two share 72% similarity, for Fe(III)–Ent uptake. PfeA is the high-affinity receptor. The crystal structure of PfeA was determined using Ent as the substrate [172]. When the substrate binds, Arg480 and Gln482 at the binding site make electrostatic/cation–π interactions with two of the Ent catechol rings. No π–π stacking interactions were observed at the binding site. The third ring is buried in the protein, shielded from solvent. In addition to Ent, PfeA can also transport the ferric complexes of azotochelin and protochelin (Figure 10) in a similar fashion [173]. Protochelin is a tris-catechol siderophore and azotochelin is a bis-catechol siderophore produced by *Azotobacter vinelandii* [174]. Two of the catechol rings in Fe(III)–azotochelin and Fe(III)–protochelin were found at the same position as two in Fe(III)–Ent in the crystal structures, suggesting that only two catechol rings are critical for substrate recognition. After entering the periplasm, it is speculated that the FepBCDG system is responsible for taking the substrate into the cytoplasm [165].

## 3. Siderophore Conjugate Studies

Because uptake pathways in Gram-negative bacteria allow them to assimilate iron, they present targets for antibiotic development. The individual proteins (i.e., TBDTs, PBPs, ABC transporters) could, in principle, represent targets in their own right. Alternatively, designed conjugates that link a toxic warhead to a siderophore—the Trojan Horse strategy—leverage the iron transport infrastructure to deliver an antibiotic to its target. Choosing the right siderophore is important because it is paramount for recognition along the transport pathway. It must also sustain structural modifications that allow the installation of the linker and warhead while enabling recognition and uptake of the conjugate.

An effective conjugate by definition requires an antibiotic warhead that can reach the desired target. Considerations involved in choosing an antibacterial warhead are (i) the mechanism of action of the antibiotic, particularly the location of its target; and (ii) the fate of the siderophore molecule in the bacterial cell. We need to first consider where the target of the antibiotic is (e.g., periplasm or cytoplasm) in order to choose the appropriate siderophore as the delivery vehicle. Different siderophores used by the same pathogen can have drastically different uptake mechanisms. For example, Pvd and Pch are both utilized by *P. aeruginosa* but each has a different landing spot. Since Pvd only reaches the periplasm of *P. aeruginosa*, a Pvd conjugate with a warhead that engages a cytoplasmic target will most likely be ineffective as the antibiotic will be less likely to be found there. Instead, Pch, on the other hand, is a good candidate to deliver a drug with a cytoplasmic target for inhibition. A Pch conjugate containing a drug with a periplasmic target has antibacterial potential but will most likely require a labile linker for early release in the periplasm.

Finally, a linker, though not always indispensable, can facilitate the synthesis of the siderophore–drug conjugate and also influence the potency of the antibiotic warhead. The site of linkage on both the siderophore and the warhead has to allow for interaction with the important machinery of the function of each. A labile linker that allows the release of a warhead at a desired location is a valuable way of addressing at least part of this concern. A warhead that, for example, inhibits a target in the cytoplasm, might lose its antibacterial activity if it experiences an early release in the periplasm. At the same time, a stable linker that prevents the release of the warhead can limit the interactions between the warhead and its target and therefore attenuate the antibacterial activity.

**Figure 11:**
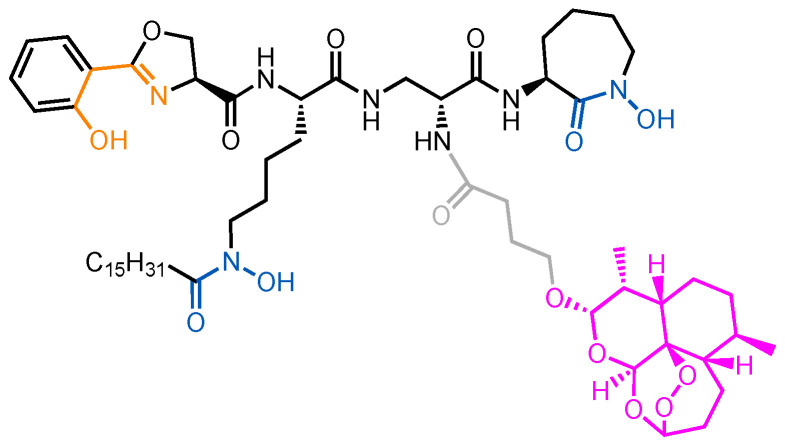
Structure of a mycobactin–artemisinin conjugate (blue: hydroxamate, orange: other iron-chelating moieties, pink: warhead).

The role of each component is best demonstrated through an example of a highly successful synthetic sideromycin. A mycobactin–artemisinin conjugate (Figure 11) inhibits two of the deadliest pathogens, *Mycobacterium tuberculosis* and *Plasmodium falciparum* [175]. The siderophore bears two hydroxamates and a hydroxyphenyl–oxazoline moiety that, together, bind Fe(III). Its warhead is artemisinin, an antimalarial natural product discovered in the 1970s [176], attached via an amide bond. Artemisinin acts by generating free radicals in cytoplasm via the reduction of Fe(III) [175]. Despite access to artemisinin as a routine treatment for malaria, *P. falciparum* remains one of the deadliest pathogens known, causing an estimated 608,000 deaths in 2022 [177]. Artemisinin on its own does not inhibit *M. tuberculosis*, likely due to a lack of uptake by the pathogen. Nevertheless, the mycobactin–artemisinin conjugate was potent against not only multiple strains of *P. falciparum* (IC_50_ = 0.0040–0.0051 μg/mL) but also *M. tuberculosis* (MIC = 0.16–1.25 μg/mL) [175]. The potency of the conjugate against *M. tuberculosis* suggested that the warhead was active in the cytoplasm.

**Figure 12:**
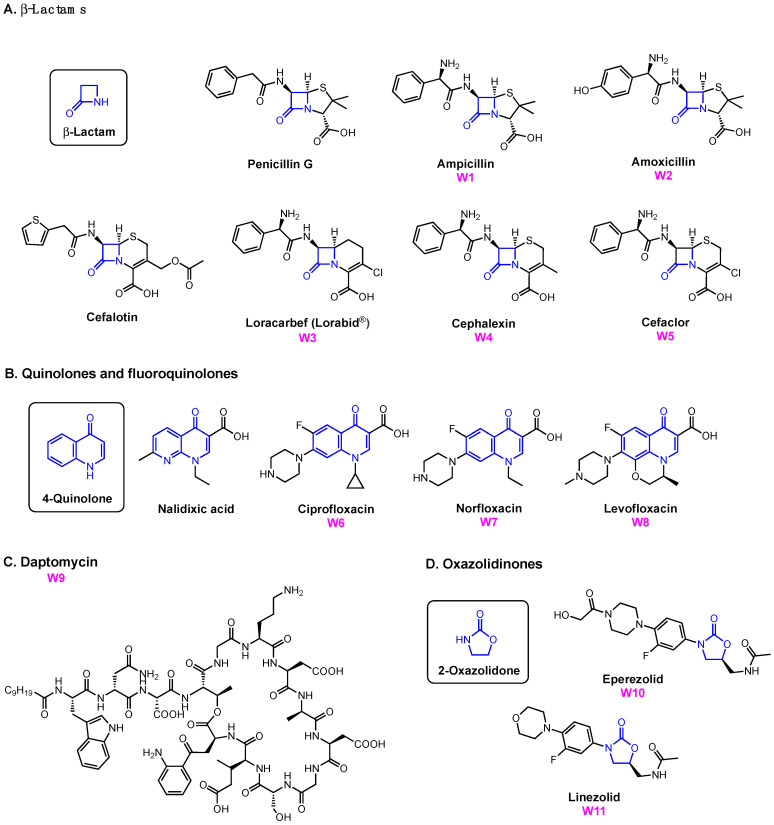
Warheads employed in Trojan Horse siderophore conjugates (blue: core motif).

A growing library of synthetic sideromycins has been designed by combining various siderophores and warheads. In the following sections, sideromycins based on the archetypical siderophores, Fim from *A. baumannii*, Ent from *E. coli*, and Pch from *P. aeruginosa*, will be treated as case studies. Other catecholate and hydroxamate siderophore–drug conjugates potent against the three pathogens will also be presented. Among them, a number of warheads were employed to inhibit various cellular targets (Figure 12). These warheads include (A) β-lactams that inhibit bacterial cell wall synthesis by binding to penicillin-binding proteins in the periplasm [178]; (B) fluoroquinolones for inhibiting DNA gyrase and topoisomerase IV involved in bacterial DNA synthesis in the cytoplasm [179,180,181,182]; (C) daptomycin, a lipopeptide antibiotic with a poorly understood mode of action that is structurally similar to cationic antimicrobial peptides that are known to disrupt the function of bacterial cell membranes and leads to disruption of DNA, RNA, and protein synthesis [183,184]; and (D) oxazolidinones that target the 50S ribosomal subunit and consequently inhibit protein synthesis [185]. Gram-negative bacteria have slowly built up antibacterial resistance by, for example, expressing β-lactamases to hydrolyze β-lactam drugs [186,187] and mutating native proteins such as gyrase or topoisomerase IV to protect themselves from fluoroquinolones [179,188,189,190,191,192]. Other antibiotics such as daptomycin and oxazolidinones are known to be primarily effective against only Gram-positive bacteria due to their inability to permeate the Gram-negative outer membrane. Siderophore conjugates containing these warheads have proved to be effective in inhibiting Gram-negative bacteria, including *A. baumannii*, *E. coli*, and *P. aeruginosa*. Their enhanced antibacterial activity stems from the active transport via different siderophore uptake pathways. The potency of conjugates carrying daptomycin or an oxazolidinone was due to the active transport these conjugates go through to bring the warhead into the bacterial cell. As a result, the impermeability of the Gram-negative outer membrane was no longer an issue for the parent drugs. In the meantime, siderophore conjugates that were ineffective against the three pathogens will be presented to suggest why they may have failed based on the location model of Trojan Horse design.

In the sections that follow, we present siderophore–drug conjugates based on the identity of the siderophore. Albomycin is presented in more detail as the prototypical Trojan Horse sideromycin, and then fimsbactin A, enterobactin, and pyochelin are used as the archetypical siderophores that have been linked to antibiotic warheads. The resulting siderophore conjugates were tested against representative strains of *A. baumannii*, *E. coli*, and *P. aeruginosa* to assess their antibacterial activity and the relationship between their potency and the location of their inhibitory target in each bacterial cell. The aim is to tease out design principles from these examples that can be used as the guideposts for the development of new conjugates with the intention of inhibiting a specific cellular target of a given pathogen. To do that, we present these siderophore–drug conjugates and their biological activities using heat maps to draw the connections between their structure, the corresponding siderophore transport machinery, and their targets. We re-define the potency of these siderophore–drug conjugates based on the results of various studies, using a color-coding system. High-potency conjugates, highlighted in dark blue, showed (i) a minimum inhibitory concentration (MIC) of less than 2 μM or 1 mg/L; (ii) an MIC_50_ of less or equal to 0.1 μM; and (iii) a growth inhibition zone greater than 30 mm. Medium-potency conjugates, highlighted in medium blue, instead had (i) MICs ranging from 2 to 20 μM or 1 to 20 mg/L; (ii) an MIC_50_ of 0.1–0.3 μM; or (iii) a growth inhibition zone between 15 and 30 mm. Finally, low-potency conjugates, highlighted in light blue, were defined as having (i) MICs of 20–100 μM or 20–150 mg/L; (ii) an MIC_50_ of 0.3–1.0 μM; or (iii) a growth inhibition zone between 5 and 15 mm. Inactive conjugates are highlighted in grey. Based on the system we have constructed here, the siderophore–drug conjugates that will be discussed in the following sections are defined as highly potent, moderately potent, or slightly potent.

### 3.1. Albomycin and Hydroxamate Siderophore Conjugates

**Figure 13:**
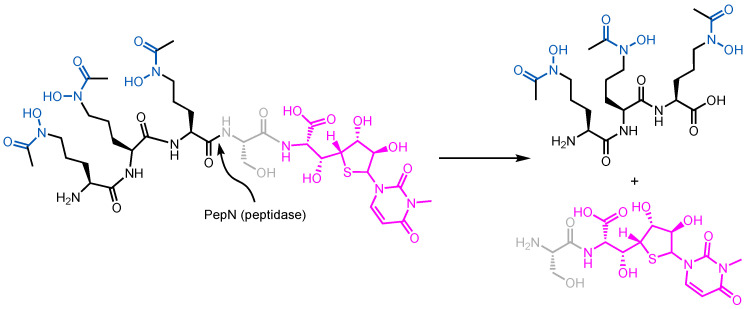
Site of cleavage of albomycin liberation of nucleoside antibiotic (blue: hydroxamate, grey: linker, pink: warhead).

As a bona fide sideromycin natural product, albomycin is the prototypical Trojan Horse antibiotic (Figure 13). Its hydroxamate motif allows it to utilize Fch’s Fhu pathway to enter *E. coli*. This is supported by the crystal structure of FhuA with albomycin in the substrate binding pocket where albomycin interacts with the same key residues involved in Fch recognition [92]. After crossing the outer membrane through FhuA, albomycin is then chaperoned by FhuD in the periplasm and passes the inner membrane through FhuCB. In the cytoplasm, Fe(III) in the complex is first reduced and then the peptide bond in the D-serine linker is hydrolyzed by peptidase N (PepN), liberating the warhead so that it can engage the seryl-tRNA synthetase in the cytoplasm [193]. This example provides insights into the keys to a successful sideromycin. For one, the siderophore moiety takes part in substrate recognition and transport by interacting with protein infrastructure in a fashion that is essentially identical to the siderophore. These interactions include (i) bonding interactions (e.g., π–π, hydrogen bonding) with complementary residues and/or (ii) electrostatic interactions between the substrate and a charged cluster in the substrate binding site. For another, the choice of linker is case-specific. An appropriate linker should be cleaved at a location that will not impede the antibiotic action of the warhead. Ideally, a warhead for an intracellular target should be cleaved in the cytosol, though this may not be essential. Release of the warhead early—in the periplasm—can lead to two outcomes. One, the warhead completely loses its antibacterial activity due to the lack of interactions with its target. Alternatively, the warhead crosses the inner membrane by passive diffusion without being destroyed or eliminated through efflux to inhibit its target. At the same time, a stable linker may attenuate the antibacterial activity of the warhead due to the lack of proper engagement of its target. In other words, the linker should be chosen and adjusted accordingly and may require a trial-and-error approach.

**Figure 14:**
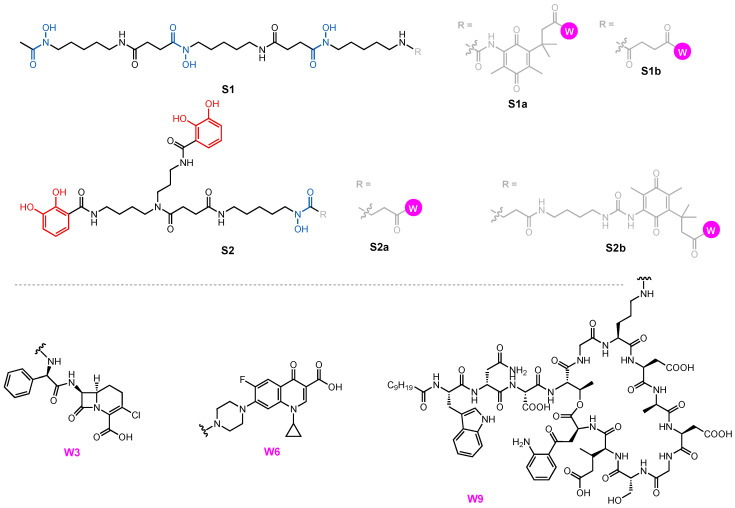
Hydroxamate and fimsbactin conjugates used in antibiotic studies (red: catecholate, blue: hydroxamate, grey: linker, pink: warhead).

Among the three Gram-negative pathogens, only *E. coli* uses hydroxamate siderophores as a major pathway for iron uptake. FpvB has, however, been shown to transport Fch and DFB in *P. aeruginosa* [158]. The major siderophores of *A. baumannii* and *P. aeruginosa*, PreAcb, Acb, Pvd, and Pch, are all mixed-type siderophores. Nevertheless, we present two hydroxamate sideromycins, **S1a-W6** and **S1b-W6**, each carrying a ciprofloxacin (**W6**) warhead (Figure 14) [194]. **S1a** contains a cleavable ‘trimethyl lock’ linker (vide infra) and **S1b** contains a stable succinic amide linker. Using an agar diffusion antibacterial susceptibility assay, the two conjugates were tested against the wild-type strains of *E. coli* and *P. aeruginosa*, and *Pseudomonas* mutant K799/61, which is highly susceptible to antibiotics such as β-lactams [143]. Based on their structures, we speculate that **S1a-W6** and **S1b-W6** enter *E. coli* using FhuE and *P. aeruginosa* via FvpB. **S1a-W6**, which possesses a redox-sensitive linker, was significantly more potent than **W6** and **S1b-W6** when tested against wild-type *P. aeruginosa* (Table 2, Entry 3). Nevertheless, this trend was not observed when **S1a-W6** and **S1b-W6** were tested against *P. aeruginosa* K799/61 and *E. coli* where the two conjugates showed comparable potency. A lack of enhanced antibacterial activity of **S1a-W6** might be due to the fact that *P. aeruginosa* K799/61 and *E. coli* cannot recognize the linker, cleavable or not. In addition, **S1a-W6** and **S1b-W6** showed no enhanced inhibitory effect against *E. coli* compared to **W6** on its own, suggesting competition between the reduction of Fe(III) versus the linker, therefore limiting the release of the warhead. In other words, the effect of a cleavable linker on the potency of a siderophore–drug conjugate might be organism- and strain-specific. In the same study, **S2a-W6** and **S2b-W6** based on a bis-catecholate mono-hydroxamate siderophore showed a different activity profile and will be discussed in the next section [194].

### 3.2. Fimsbactin conjugates

While siderophore–drug conjugates of Fim that target *A. baumannii* have not been reported due to their recent discovery, synthetic mimics of Fim have proved to be reliable surrogates as a part of conjugates to inhibit *A. baumannii*. Bis-catecholate mono-hydroxamate siderophore **S2a** (Figure 14), for example, was reported by Miller well before the discovery and isolation of fimsbactins [195]. The structure of **S2a** resembles Fim such that conjugate **S2a-W3** was evaluated as a Trojan Horse against *A. baumannii*, *E. coli*, and *P. aeruginosa* [70,196]. The antibacterial activity of loracarbef (**W3**) and conjugate **S2a-W3** against the three organisms is summarized in Table 3. **W3** is a β-lactam that targets the penicillin-binding proteins in the periplasm, so **S2a-W3** only needs to penetrate the outer membrane to reach its target. **W3** on its own was potent against *E. coli* (MIC = 2 μM) but inactive against *A. baumannii*. Conjugate **S2a-W3**, on the other hand, was active against *A. baumannii* (MIC = 0.125 μM) but less potent than **W3** against *E. coli* (MIC = 8 μM). *P. aeruginosa* was insensitive to both **W3** and **S2a-W3**. The enhanced potency of **S2a-W3** against *A. baumannii* could be due to the utilization of the Fim pathway to penetrate the outer membrane. This was supported by the finding that the inhibitory effect of **S2a-W3** could be antagonized by the addition of **S2a**, suggesting a competition for the uptake by a specific pathway. The *P. aeruginosa* siderophores Pvd and Pch share few structural similarities with Fim and **S2a**. Although Pvd is also a mixed-type siderophore that binds Fe(III) via catecholate and hydroxamate moieties, the high selectivity of FpvA for Pvd of each *P. aeruginosa* strain probably prevents the uptake of Fim and related analogs.

Prompted by the activity of **S2a-W3** against *A. baumannii*, two additional conjugates were investigated by exchanging the β-lactam **W3** for fluoroquinolone **W6**. In the same study on **S1a-W6** and **S1b-W6**, **S2a-W6** and **S2b-W6** were also employed. **S2b-W6** contains the labile quinone ‘trimethyl lock’ linker that can undergo reduction to release the warhead (Figure 14) [194]. As expected, **S2b-W6** was indeed more potent than **S2a-W6** due to its ability to release the warhead. Both **S2a-W6** and **S2b-W6** were able to inhibit *E. coli*, *A. baumannii*, and *P. aeruginosa*, and **S2b-W6** was the most potent against all three strains (Table 2). Only **W6** and **S2b-W6** were able to inhibit *P. aeruginosa* K799/61, with **S2b-W6** showing markedly lower potency than **W6**. The active transport of **S2b-W6** and the labile linker did not give this conjugate any advantage against *P. aeruginosa* K799/61. However, **S2a-W6** and **S2b-W6** (Dia. ≥ 18 mm) showed higher potency than **W6** only when tested against *A. baumannii* (Table 2), suggesting that the conjugates have a higher affinity for the siderophore transport system in *A. baumannii*, possibly the Fim pathway. Overall, fimsbactin-inspired conjugates **S2a-W6** and **S2b-W6** were less potent than hydroxamate conjugates **S1a-W6** and **S1b-W6.** With respect to fluoroquinolone warhead **W6**, both conjugates only showed enhanced antibacterial activity against *A. baumannii*, whereas their potency was noticeably reduced when tested against *E. coli*, and *P. aeruginosa*. This is consistent with the behavior of **S2a-W3** where *A. baumannii* was able to recognize and transport **S2a** with the greatest efficiency. Fiu and CirA in *E. coli* might be able to transport **S2a** but not as efficiently. Compared with the complete lack of inhibition by **S2a-W6**, *P. aeruginosa* K799/61 was susceptible to **S2b-W6**. This difference suggests that either this strain does not recognize and transport **S2a-W6**, or that the attenuated antibacterial activity of **S2a-W6** was simply due to the lack of release of the warhead. The study highlights the significance of a linker that can liberate the warhead inside the cell, allowing the subsequent inhibition. Although the significance of a labile linker might vary in different strains of the same species, for example, wild-type *P. aeruginosa* and mutant strain K799/61, it is worth noting that a linker is sometimes the key to enhancing the potency of a siderophore–drug conjugate.

Daptomycin conjugate **S2a-W9** (Figure 14) utilized the fimsbactin surrogate to smuggle a warhead into the periplasm of *A. baumannii* and inhibited ten different strains of *A. baumannii* with MICs ranging from 0.4 to 0.8 μM (Table 4) [197]. It was previously shown that daptomycin was inactive against *P. aeruginosa* and *E. coli* because of its inability to permeate their outer membranes and also the inability to disrupt their cytoplasmic membrane [198]. Despite the presumably successful transport of **S2a-W9** into *E. coli* and *P. aeruginosa*, daptomycin lacks the ability to inhibit the two pathogens. Another daptomycin-containing conjugate **S4-W9** using an analog of Ent as the siderophore will be discussed later.

**S2a-W3**, **S2a-W6,** and **S2a-W9**, each with a distinct warhead for a unique cellular target, showed antibacterial activities against *A. baumannii*. All three conjugates showed higher potency than their corresponding drug conjugate alone, suggesting that uptake was important for activity. **S2a-W3**, **S2a-W6**, and **S2b-W6** were also able to inhibit *E. coli*, possibly via the action of Fiu and CirA, although with reduced potency than the parent drugs. The conjugate that showed the highest potency against *E. coli*, **S2b-W6,** contained a redox-active linker that allows the intracellular release of **W6**. Only **W6**-containing conjugates afforded inhibition against wild-type *P. aeruginosa* and K799/61. We speculate that there is a limited number of siderophore transport systems in *P. aeruginosa* that are compatible with **S2a** or **S2b** and can enable sufficient uptake of the conjugates. **W3** and **W9**, which both act in the periplasm, might not have been active warheads for *P. aeruginosa* or required a cleavable linker for release in *P. aeruginosa*. **W6**, on the other hand, acts in the cytoplasm. The early release of **W6** from the conjugate might still allow the passive diffusion of the warhead into the cytoplasm to inhibit its target. Interestingly, **W9** was active against *A. baumannii* only, and this is consistent with previous reports revealing that *E. coli* and *P. aeruginosa* might lack the target for daptomycin [198].

### 3.3. Enterobactin Conjugates and Derivatives

**Figure 15:**
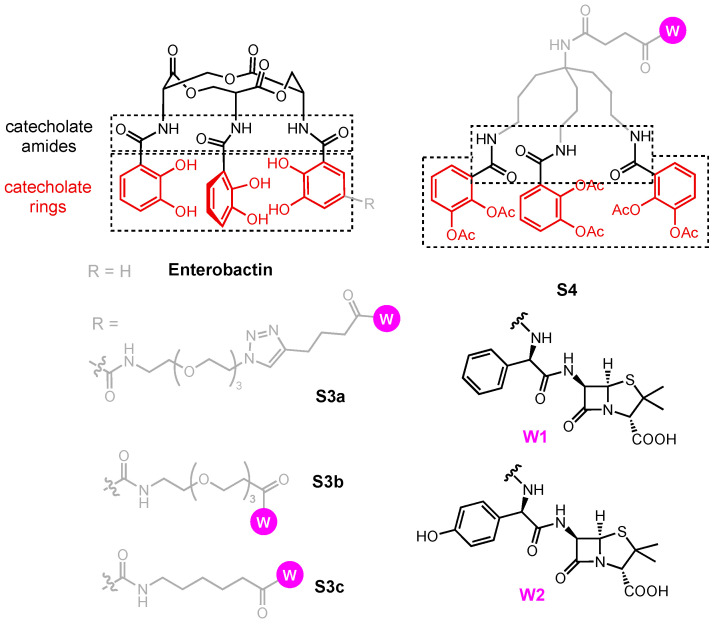
Conjugates of enterobactin and related derivatives used in antibiotic studies (red: catecholate, grey: linker, pink: warhead).

Enterobactin (Ent) is the primary endogenous siderophore used by *E. coli* and used as a xenosiderophore by *P. aeruginosa*. Due to its prevalence and its affinity for Fe(III), Ent has been the subject of extensive studies on siderophore conjugates. Siderophore **S3a** (Figure 15) is derived from Ent and contains a stable, water-soluble PEG_3_–triazole linker installed on a catecholate ring [199]. Mimicking the design of salmochelins, the linker was placed at the C5 position and was coupled to ampicillin (**W1**) and amoxicillin (**W2**). Conjugates **S3a-W1** and **S3a-W2** and the parent β-lactams **W1** and **W2** were tested against wild-type *P. aeruginosa* and six strains of *E. coli* (Table 5). **W1** and **W2** were inactive against *P. aeruginosa* (MIC >100 µM). However, both **S3a-W1** and **S3a-W2** showed inhibition against *P. aeruginosa* (MIC = 10 µM). *P. aeruginosa* expresses two Ent-transporting outer membrane receptors, PfeA and PirA, responsible for the uptake of **S3a-W1** and **S3a-W2**. Under iron-deficient conditions, **S3a-W1** and **S3a-W2** were 10- to 100-fold more potent against *E. coli* 25922, UTI89, H9049, and 43895 than **W1** and **W2**. For *E. coli* CFT073, a strain that expresses multiple Ent uptake pathways, MIC values as low as 10 nM were recorded under iron-deficient conditions but **S3a-W1** and **S3a-W2** showed no enhanced antibacterial activity against *E. coli* 35401. Gram-negative bacteria repress the production of siderophores and the corresponding transport proteins under iron-rich conditions [200]. These results indicate that production of the siderophore transport system responsible for the uptake of **S3a-W1** and **S3a-W2** was present and active under iron-poor conditions, leading to an enhanced uptake of the conjugates.

To confirm if the antibacterial activity of **S3a-W1** and **S3a-W2** arose from the β-lactams, hydrolyzed analogs were tested against wild-type *E. coli*; these compounds retained siderophore activities but no inhibition of growth was observed. Additional insight into the uptake of **S3a-W1** and **S3a-W2** employed three knockout strains of *E. coli* K-12: *fepA*- (TBDT knockout), *fepC*- (ABC Transporter ATPase knockout), and *fes*- (hydrolase knockout). In *E. coli* K-12, MICs of 0.1 µM were recorded for both conjugates under iron-deficient conditions (Table 5, Entry 8). The hypothesis was that **S3a-W1** and **S3a-W2** should have reduced potency against *fepA*- due to iron starvation and the results were as expected where both **S3a-W1** and **S3a-W2** experienced a reduction in their antibiotic activity (Table 5, Entry 9). The potency of **S3a-W1** and **S3a-W2** was similar to the parent drug against *fepA*-. Without active uptake, the growth inhibition observed was solely due to a lack of iron supply to *fepA*-. The growth of *fepC*- and *fes*- was inhibited by **S3a-W1** or **S3a-W2** under iron-deficient conditions (Table 5, Entries 10 and 11), similar to what was observed for wild-type *E. coli* K-12. Accumulation of **S3a-W1** or **S3a-W2** inside the cells of *fepC*- and *fes*- did not lead to any noticeable improvement in the antibacterial activity of the conjugates, possibly due to the competition between penicillin-binding proteins and other PBPs such as FepB. Overall, **S3a-W1** and **S3a-W2** were reasonably potent against *E. coli* and *P. aeruginosa* under iron-poor conditions. Nolan demonstrated that **S3a-W1** and **S3a-W2** utilized FepA in *E. coli* and possibly PfeA and PirA in *P. aeruginosa* to inhibit their periplasmic targets. Here, we speculate that other similar Ent conjugates utilize the same pathway to enter *E. coli*. In addition, the C5 position of the catechol ring was a suitable site for bearing a linker which did not interfere with FepA uptake.

Following the established **S3a** design, Ent–ciprofloxacin conjugates **S3b-W6** and **S3c-W6** were also studied. **S3b** carries a PEG_3_ linker whereas **S3c** possesses a short aliphatic linker. They are similarly stable but vary in length. **S3b-W6** and **S3c-W6** were initially reported to be inactive against *E. coli* K-12 and *P. aeruginosa* [201], but later testing against non-pathogenic *E. coli* K-12 and uropathogenic strains *E. coli* UTI89 and CFT073 showed them to be potent against the latter two strains (MIC = 0.1–1 μM) (Table 6, Entries 1 and 2) [202]. Under iron-rich conditions, the antibacterial activity of **S3c-W6** against *E. coli* CFT073 was suppressed, but against *E. coli* UTI89, it was not affected. In addition to Ent, uropathogenic strains of *E. coli* also produce salmochelin (Figure 8) and express the TBDT IroN [203,204,205]. Tests on mutant strains *fepA-*, *iroN-*, *fepA-/iroN-*, *fepC-*, and *fepDG-* revealed the uptake mechanism of **S3c-W6**. Compared to wild-type, the antibacterial activity of **S3c-W6** was retained when tested against *fepA-* and *iroN-* (Table 6, Entry 5 and 6) and was attenuated against the double mutant f*epA- iroN-* (Table 6, Entry 7), suggesting that **S3c-W6** used both FepA and IroN to enter the periplasm. When tested against *fepC-* and *fepDG-*, the antibacterial activity of **S3c-W6** was completely lost (Table 6, Entries 8 and 9), suggesting it was transported by FepCDG into the cytoplasm. Tests on *fes- and iroD-*, mutants lacking the hydrolase for breaking down Ent and salmochelins, respectively, demonstrated the fate of **S3c-W6** in the *E. coli* cytoplasm [133]. Despite the fact that the transport mechanism of **S3c-W6** is mostly in complete alignment with the *E. coli* Ent pathway, the release of the warhead does not rely on Fes, but rather IroD (Table 6, Entry 10 and 11). This finding is surprising but more than reasonable as salmochelins are in essence Ent derivatives. Moreover, this might be the key to justify the lack of antibacterial activity of **S3b-W6**. As both **S3a** and **S3c** utilize the Ent pathway to enter *E. coli*, it is intuitive to hypothesize that **S3b** follows the same mechanism. We thus rule out the possibility that **S3b-W6** was not able to enter the *E. coli* periplasm. Instead, the long, flexible PEG_3_ linker in **S3b-W6** might have prevented the conjugate from crossing the inner membrane through FepCDG. Alternatively, it could have also prevented the interactions of **S3b-W6** with IroD and the consequent release of **W6**.

Following another strategy, Miller prepared a siderophore that replaced the trilactone ring of Ent with an acyclic core (Figure 15, **S4**) [206]. According to previous studies, the key structural motifs involved in Ent recognition were the catechol rings and the adjacent amide groups [207]. **W1**, **W2**, **S4-W1**, and **S4-W2** were tested against *E. coli* and five strains of *P. aeruginosa* (Table 5). **W1** and **W2** were highly potent against the permeable strain *P. aeruginosa* K799/61 (MICs = 0.39–0.78 μM) and moderately active against *E. coli* (MICs = 4.17–16.7 μM). The remaining strains were insensitive to both **W1** and **W2**. Under iron-rich conditions, **S4-W1** and **S4-W2** were mildly active against all strains (Table 5, Entry 1, 2, 12–14) except the clinical isolate *P. aeruginosa* Pa6 (Table 5, Entry 15). Under iron-deficient conditions, though, they were potent against strains of *P. aeruginosa* (MICs = 0.05–0.39 μM) with the exception of *P. aeruginosa* Pa6. *P. aeruginosa* Pa6 was completely resistant to **W1**, **W2**, **S4-W1**, and **S4-W2** regardless of iron availability. **S4-W1** and **S4-W2** were also active against *E. coli* but with reduced potency (Table 5, Entry 2, MICs = 1.56, 6.15 μM). The difference in the antibacterial activities of **S4-W1** and **S4-W2** against *E. coli* and *P. aeruginosa* highlighted differences in their Ent transport systems. **S4-W9** containing a daptomycin warhead showed similar activity to **S2a-W9** where **S4-W9** was only able to inhibit *A. baumannii* (Table 4, Entry 1,2, 7–10) whereas *E. coli* and *P. aeruginosa* were both resistant (Table 4, Entry 12, 14, 15) [208]. Based on the ability of *E. coli* and *P. aeruginosa* to transport **S4-W1** and **S4-W2**, **S4-W9** further illustrates that **W9** is most likely simply ineffective against the two pathogens. Furthermore, *A. baumannii* is able to utilize **S4** in addition to PreAcb, Acb, and Fim. Compared with **S2a-W9**, **S4-W9** was less potent against the six strains of *A. baumannii* tested. As a closer analog of Fim, **S2a** may simply be a more compatible substrate for the Fim uptake pathway in *A. baumannii*. As a result, the enhanced uptake of **S2a-W9** led to greater antibacterial activity than **S4-W9**. Nevertheless, **S4** is no doubt a viable starting point for siderophore–drug conjugates targeting *A. baumannii*, *E. coli*, and *P. aeruginosa*.

### 3.4. Catecholate Siderophore Conjugates

**Figure 16:**
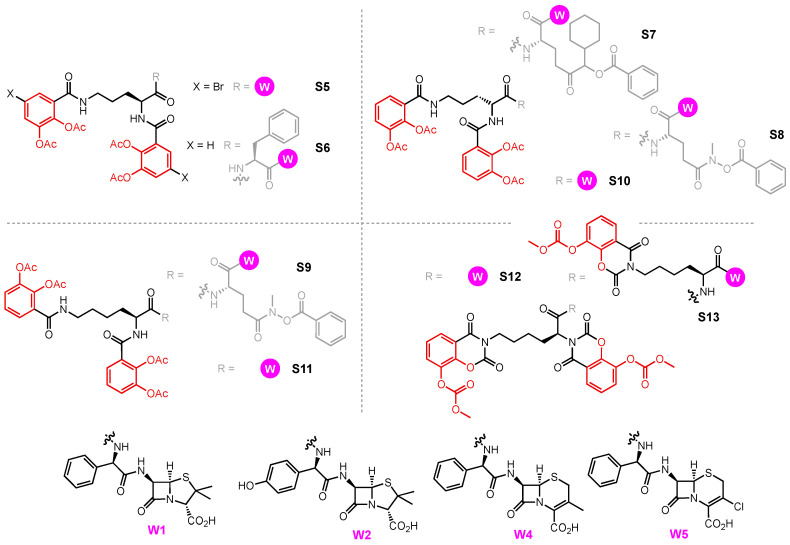
Catecholate conjugates used in antibiotic studies (red: catecholate, grey: linker, pink: warhead).

Regardless of the natural product that inspired their design (i.e., Ent, Fim, etc.), other catecholates have also been evaluated as siderophore conjugates. Heinisch and colleagues reported a series of bis- and tris-catecholate siderophores linked to a β-lactam antibiotic (Figure 16, **S5**-**S13**) [209]. They likely enter *E. coli* via the TBDTs Fiu and Cir, and *P. aeruginosa* via PfeA. In each, the catecholate groups were acylated to prevent unwanted methylation in vivo. Without prior complexation to Fe(III), an acylated catecholate siderophore acts as a prodrug [210,211]. Conjugates carrying the **W1** warhead, including **S5-W1**, **S6-W1**, **S7-W1**, **S8-W1**, and **S9-W1** (Table 7, Entry 3) were potent against *P. aeruginosa* (MICs ≤ 0.05 mg/L). They exhibited an approximate 10-fold decrease in potency against *E. coli* (MICs = 0.1–0.78 mg/L) (Table 7, Entry 1) and *P. aeruginosa* ATCC 27853 (MICs = 0.2–0.78 mg/L) (Table 7, Entry 2). Differences in size, structure, and stereochemistry in **S5-W1, S6-W1, S7-W1, S8-W1,** and **S9-W1** did not influence the antibacterial activity of each conjugate. The activity of conjugates **S12-W1** and **S13-W1** was variable. The **W4** conjugates **S10-W4** and **S11-W4** had poorer activity; it is uncertain if this was attributable to the warhead or the siderophore. The lack of inhibition against *E. coli* suggested that the antibiotic activity of **S10-W4** and **S11-W4** might be strain-specific or **W4** might not be a suitable warhead for it. Last, **S13-W1**, **S13-W2**, and **S13-W5** relied on the same siderophore but contained a different β-lactam antibiotic in each structure. **S13-W1** (MIC = 0.78, 0.2 mg/L) and **S13-W2** (MIC = 6.25, 0.78 mg/L) were moderately to highly potent against strains of *P. aeruginosa*, whereas **S13-W5** showed significantly reduced potency (MIC = 50 mg/L). The different potencies suggest that the antibacterial activity of these conjugates was due to the action of the β-lactam instead of iron starvation. It is worth noting the halogen in the catechol C5 position of **S5-W1**; its electron-withdrawing effect influences the electronics of the ring and its ability to chelate Fe(III). A similar structural motif is seen in cefiderocol, suggesting that the halogen likely played a role in its activity.

**Figure 17:**
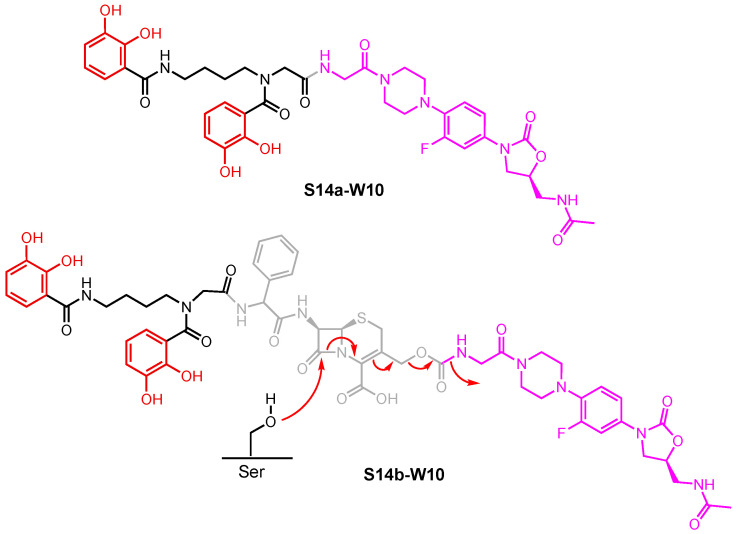
A β-lactamase activated Trojan Horse antibiotic (red: catecholate, grey: linker, pink: warhead).

While β-lactams have been primarily used as warheads in siderophore conjugates, they have also been used as linkers due to their susceptibility to β-lactamases. Previous studies showed that cephalosporins, even when they are linked to other molecules, can be hydrolyzed by cephalosporinases [212]. Miller reported on conjugates using a bis-catecholate siderophore and **W10** as the warhead (Figure 17) [213]. Two conjugates, **S14a-W10** and **S14b-W10,** were prepared to assess the effectiveness of the linker and the consequent antibacterial effect (Table 8). Neither warhead **W10** nor **S14a-W10**, containing a non-cleavable linker, showed antibacterial activity against four strains of bacteria. With the addition of the cleavable cephalosporin linker, however, **S14b-W10** was highly potent against most strains of Gram-negative bacteria tested with MIC values ranging from <0.025 to 0.4 μM. It was also active against *A. baumannii* ATCC BAA1797 but with a significant reduction in potency (Table 8, Entry 3). Upon the action of a cephalosporinase to induce the release of the oxazolidinone warhead, **W10** accumulates in the periplasm. Then, **W10** can cross the inner membrane via passive diffusion to inhibit its target in the cytoplasm. The lack of antibiotic action of **W10** against the strains of Gram-negative bacteria tested due to the impermeability of the drug was circumvented by the active transport of the siderophore–drug conjugate. This way, the siderophore actively carries the warhead through the outer membrane to circumvent the impermeability of the parent drug. The utilization of the susceptibility of cephalosporin to cephalosporinases was taken advantage of to ensure the release of the warhead at a desired location.

### 3.5. Pyochelin conjugates

**Figure 18:**
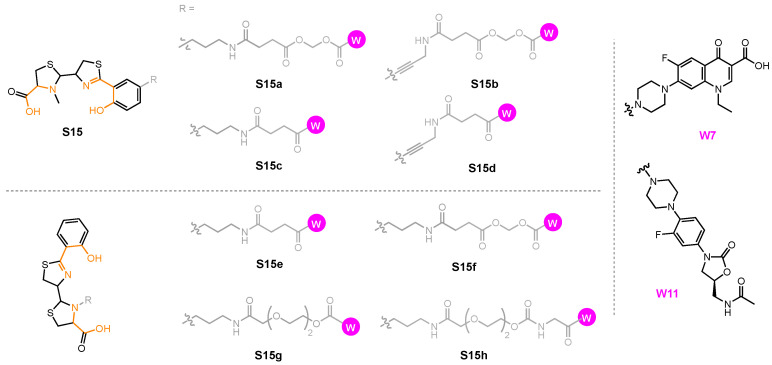
Pyochelin conjugates used in antibiotic studies (orange: other iron chelating moiety, grey: linker, pink: warhead).

**Figure 19:**
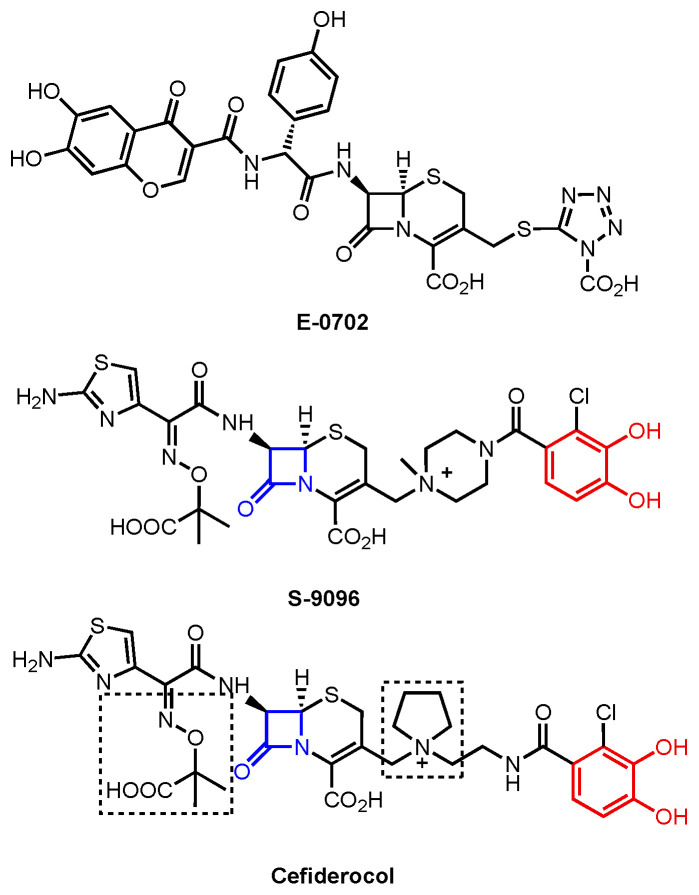
Evolution of cefiderocol via E-0702 and S-9096 (red: catecholate, blue: β-lactams).

Pyochelin (Pch) is produced by all strains of *P. aeruginosa* whereas the pyoverdins (Pvds) and their OM transporters are largely strain-specific. The potential to make conjugates that work across strains has therefore led to several Pch conjugates being reported (Figure 18) [214,215]. Among the conjugates, two sites were determined to be appropriate for functionalization: the phenol C4 position (Figure 19, **S15a**-**S15d**) and the thiazolidine N3″ position (Figure 19, **S15e**-**S15h**). The rationale behind phenol C4 functionalization originated via inspection of the Fe(III)–(Pch)_2_ complex bound by FptA [164]. In the substrate binding pocket, one phenol ring of the complex is exposed to the solvent outside the binding pocket, making it an attractive site for functionalization. The phenol C4 position in **S15a**-**S14d** was linked to norfloxacin (**W7**) through two types of linkers: labile acetal-based linkers, as in **S15a** and **S15b**, and stable succinic linkers, as in **S15c** and **S15d**. Activities of **S15a-W7**, **S15b-W7, S15c-W7**, and **S15d-W7** against *P. aeruginosa* PAO1 were consistent with a hypothesis where **S15a-W7** and **S15b-W7** (MICs = 10 μM) would exhibit antibacterial effect due to the presence of a cleavable linker [214] that allowed the warhead to engage its intracellular target after entry into the cytoplasm. **S15c-W7** and **S15d-W7** did not show any inhibitory effect against *P. aeruginosa* PAO1 at 10 μM.

**S15e** and **S15f** were functionalized at the thiazolidine N3″ position (Figure 18). A docking experiment demonstrated that the N3″ extended toward the space outside the binding pocket, making it another suitable site for modification [215]. The modified siderophore was fitted with fluoroquinolones **W6**, **W7**, and levofloxacin (**W8**). The conjugates were tested against three strains of *P. aeruginosa*: wild-type PAO1, Pvd- and Pch-deficient strain PAD07, and Pvd- and TonB-deficient strain PAD14. The three parent fluoroquinolones were highly potent against all three *P. aeruginosa* strains with MIC_50_ values ranging from 0.035 to 0.360 μM (Table 9). **S15e-W6**, **S15e-W7**, and **S15e-W8**, with stable succinic linkers, and **S15f-W8** did not exhibit any antibacterial behaviors. Only **S15f-W6** (MIC_50_ = 0.170–0.700 μM) and **S15f-W7** (MIC_50_ = 0.450–1.000 μM) showed inhibition against the three strains but with significantly lower potency than the corresponding parent drugs. Despite the fact that **S15f-W8** also contained a labile linker, its lack of antibacterial action was consistent with the attenuated potency of **W8** itself.

Pyochelin–oxazolidinone conjugates, **S15e-W11**, **S15g-W11**, and **S15h-W11**, were also inactive against *P. aeruginosa* PAO1 [216]. The three conjugates were employed using a disk diffusion assay under iron-deficient conditions and no antibacterial activity was observed. The labile linker in **S15g-W11** did not enhance the antibacterial effect of the conjugate. The lack of antibiotic action of **S15e-W11**, **S15g-W11**, and **S15h-W11** could be ascribed to a few reasons. For one, the low aqueous solubility of the conjugates at physiological pH limited the uptake by *P. aeruginosa* PAO1. For another, the Pch transport system might have failed to recognize and transport **S15e-W11**, **S15g-W11**, and **S15h-W11**. Due to the lack of detailed characterization of the Pch transport system, elements that impaired the transport of the three conjugates remain unclear. In addition, *P. aeruginosa* PAO1 could simply be resistant to **W11**.

To create siderophore–drug conjugates with other antibacterial warheads, the same design principles apply. To ensure uptake by Gram-negative bacteria, a siderophore for which the organism has a transporter is essential to uptake. It is also helpful to understand the substrate–receptor interactions that allow the siderophore to supply the pathogen with iron to determine where the appropriate site(s) for functionalization should be. Next, a warhead with a mode of action that involves a target which co-localizes where the siderophore is transported should be chosen. The knowledge of how the antibiotic inhibits its target is crucial for determining whether a labile linker is required. In other words, the correct ‘puzzle pieces’—a siderophore, a linker, and a warhead—should be tailored to the pathogen targeted. Successful siderophore–drug conjugates containing other antibiotics such as teicoplanin have been explored [217]. Similar to vancomycin, teicoplanin is a semisynthetic antibiotic that acts in the periplasmic space to inhibit bacterial cell wall synthesis but lacks inhibition against Gram-negative bacteria, presumably due to the inability to cross the Gram-negative outer membrane [218]. A siderophore–teicoplanin conjugate utilizing **S2** (Figure 14) as the siderophore was exclusively active against four strains of *A. baumannii* under iron-deficient conditions [217]. They were inactive against *E. coli* and *P. aeruginosa*, possibly due to the lack of siderophore transport pathways that were able to recognize Fim analogs such as **S2**. The conjugate did not possess a labile linker as the teicoplanin acts in the periplasm and therefore does not require linkage cleavage. However, not all large-molecule antibiotics can enter the cell this way. In Nolan’s study on enterobactin–drug conjugates, an enterobactin–vancomycin conjugate was investigated. Although the conjugate was able to inhibit the growth of *E. coli*, the antibacterial activity was attributed to iron starvation resulting from the lack of uptake, rather than the potency of the warhead [201]. It is worth keeping in mind that all three puzzle pieces—siderophore, linker, and warhead—need to fit together properly to give rise to the potency of a conjugate amid the various ways in which the puzzle pieces can be designed to fit together.

## 4. Cefiderocol

Cefiderocol is a β-lactam antibiotic approved for clinical use in the United States in 2019. Initially, β-lactams were only effective against Gram-positive organisms. Cefiderocol, however, is amongst a new list of broad-spectrum β-lactams whose action extends to Gram-negative organisms [219]. It represents the latest chapter in the long history of discovery and development of β-lactams as antibacterials for human health [220]. The origins of cefiderocol began in the 1980s with semisynthetic penicillin derivatives such as **E-0702** (Figure 19). Taking inspiration from enterobactin, moieties that carried catechol or catechol mimetics (hydroxy quinolones) were incorporated into these derivatives [211,221]. In the early 1990s, Shionogi & Co. (Osaka, Japan) applied the same rationale but used a cephalosporin framework to deliver **S-9096** [222]. This compound showed good in vitro antibacterial activity but had poor drug properties such as low substance stability and cardiovascular toxicity. Prompted by increased carbapenem resistance, the Shionogi researchers later revisited these compounds and developed cefiderocol with good antibacterial and drug properties.

A collection of several design features makes cefiderocol a successful antibiotic (Figure 19). First and foremost is the cephalosporin core containing the β-lactam unit that is responsible for its antibacterial activity. Structure–activity relationship (SAR) studies showed that the pyrrolidinium group in the C3 position is responsible for β-lactamase resistance [223,224]. Meanwhile, the carboxypropanoxyimino group at C7 facilitates outer membrane transport in Gram-negative bacteria [225]. The chloro-catechol moiety is reminiscent of enterobactin and pyoverdine and, hence, makes it a siderophore–β-lactam conjugate. The identity of the ligands that complement the catechol of cefiderocol is not clear. Enterobactin, for example, has a complete set of ligands—via three catechols—that can coordinate iron. It could be that the two carboxylates on cefiderocol could participate in coordination, but this seems unlikely. Nonetheless, a lower affinity for iron may, in fact, be important for its action in the periplasm. The penicillin-binding proteins that are the target of β-lactams are housed in the periplasm, so the siderophore antibiotic conjugates only need to cross the outer membrane to act on their targets.

Cefiderocol shows strong inhibition against *E. coli*, *P. aeruginosa,* and *A. baumannii* with MICs ranging from 0.063 to 1 mg/mL. Removal of the catechol significantly reduces antibiotic activity, providing strong circumstantial evidence for the importance of transport to its mechanism of action. Nonetheless, a correlation between antibiotic activity and affinity for iron using analogs could not be established. Studies have shown that cefiderocol makes use of CirA and Fiu in *E. coli* [226], PiuA in *P. aeruginosa* [227], and PirA in *A. baumannii* [228] to cross the outer membrane and exert its antibiotic effect. PiuA and PirA are TBDTs expressed by both *P. aeruginosa* and *A. baumannii* with slight structural variations. Their natural substrates have not been identified. In *P. aeruginosa*, PirA that transports Ent does not take part in cefiderocol uptake [228]. It has been postulated that Cir and Fiu may be receptors for the products of hydrolysis of enterobactin, essentially a recycling uptake system for its components [111]. This is a plausible mechanism that does not rely on the iron transport mechanism but does reveal that metabolite re-uptake is a related and potentially important Trojan Horse strategy of its own.

## 5. Conclusions

A variety of siderophores and the corresponding protein machinery for their active transport into the Gram-negative bacteria *A. baumannii*, *E. coli*, and *P. aeruginosa* have been presented as a context for understanding the Trojan Horse approach to antibiotic development. We have illustrated the properties of the siderophores and the protein transporters that give rise to specificity in substrate–receptor recognition including, for example, electrostatic and hydrophobic interactions, and complementary hydrogen bonding interactions between a substrate and receptor. We also highlighted gaps in the literature that should inspire future studies to reveal more about each siderophore uptake pathway. As Table 1 illustrates, some uptake pathways still lack identified transporters. For example, the fate of Pch in *P. aeruginosa* remains a mystery despite being a promising siderophore for siderophore–drug conjugate development. Being able to shine more light on the Pch uptake pathway will in turn tell us more about what is needed from Pch conjugates that can bring a warhead to a desired location to target a given strain of *P. aeruginosa*. The interplay between fundamental characterization of iron transport pathways and their exploitation in Trojan Horse approaches will continue to the benefit of both areas of research.

The fact that a given organism can produce multiple endogenous siderophores, each with its own uptake pathway, and also receptors for exogenous siderophores gives rise to an open question: What set of environmental conditions gives rise to the utilization of one iron transport pathway over another? Similarly, why do certain siderophores contribute to virulence while others do not? Being able to answer these questions will be key to understanding Gram-negative pathogens and developing Trojan Horse conjugates to combat them.

Based on the current knowledge of siderophore transport systems and the siderophore conjugates reported in the literature, parameters that are likely to deliver success have emerged. Our analysis included cases where siderophore conjugates were ineffective as antibiotics. Having these case studies in hand, a list of design principles to demonstrate what it takes for a sideromycin to be potent has been assembled. First and foremost, we emphasize ‘location’ as the key element for designing a conjugate. That means choosing a siderophore that will deliver a warhead to, or near, the location of the warhead’s target, whether it be the periplasmic space or the cytoplasm of Gram-negative bacteria. If the warhead is sequestered in a location that lacks its target, it will be ineffective. Second, the details of the linker are critical. To ensure that substrate recognition takes place, the linker has to be installed at a place that does not interfere with the siderophore and the proteins involved in its transport. It concurrently requires that the linker does not affect the engagement of the target by the warhead. Further, whether or not a linker should be cleavable depends on how well the localization of the siderophore and the warhead are matched. Warheads that can act in the periplasm, including β-lactams and daptomycin, do not necessarily require a cleavable linker to exert their antibacterial effect. Warheads with intracellular targets such as fluoroquinolones generally need a cleavable linker as a prerequisite for their antibiotic action. Upon linker cleavage, the warhead accumulates in the periplasm and crosses the Gram-negative inner membrane by passive diffusion to inhibit its target. Last, there are two additional lessons to be learned from the success of cefiderocol. They include a minimalist approach to the siderophore moiety that enables assessment of linkage strategies. It also points to re-uptake pathways for key metabolites, like siderophore building blocks, as areas for further inquiry and development of Trojan Horse strategies.

A potential drawback of the strategy is that, despite their potential to circumvent antibacterial resistance, siderophore–drug conjugates can be challenging to develop. The need to fit the correct siderophore, linker, and warhead together to ensure delivery of the conjugate to the desired destination is often conducted by trial and error. Moreover, activity in in vitro investigations is just the first of many steps in the multi-step process of drug development. Subsequent steps include demonstration of in vivo activity and favorable pharmaceutical properties (solubility, PK/PD, toxicity, etc.), all of which carry their own challenges.

Overall, it is important to take the following questions into consideration when designing a new siderophore conjugate: (i) Which pathogen is being targeted and what siderophore(s) does the species use? (ii) What is the uptake mechanism and fate of the chosen siderophore and how does it interact with the corresponding protein transporters? (iii) Where is the appropriate modification site to install a linker and a warhead that will not hinder productive intermolecular interactions? (iv) Are the siderophore and warhead matched in terms of location (periplasm or cytoplasm)? Being able to answer these questions will position future studies for success in the development of active siderophore conjugates.

## Figures and Tables

**Table 1:** Key protein receptors involved in siderophore transport systems in *A. baumannii*, *E. coli*, and *P. aeruginosa*.

	*A. baumannii*	*E. coli*			*P. aeruginosa*	
	(Pre)acinetobactin (Pre)Acb	Ferrichrome Fch	Enterobactin Ent	Catecholate Siderophores	Pyochelin Pch	Enterobactin Ent
**Outer membrane** **(TBDT)**	BauA	FhuA	FepA IroN	Fiu CirA	FptA	PfeA PirA
**Periplasm** **(PBP)**	BauB	FhuD	FepB	Unknown ^?^	FepB ^?^	FepB ^?^
**Inner membrane** **(ABC)**	BauCDE	FhuCB	FepDGC	Unknown ^?^	FptX FepDGC PchHI	FepDGC ^?^
**Cytoplasm**	BauF *	FhuF *	Fes ^#^ YgjH *	Unknown ^?^	Unknown ^?^	Unknown ^?^

Notes: * reductase; ^#^ esterase; ^?^ unidentified/unknown/speculated.

**Table 2:** Antibiotic activity of **S1** and **S2** conjugates determined by diameter of growth inhibition zone (mm).

Entry	Strain	W6	S1a-W6	S1b-W6	S2a-W6	S2b-W6
1 ^a^	*A. baumannii* ATCC 17961	15 ^b^	-	-	18	20
2	*E. coli* X580	31 ^c^	34	32	21	27
3	*P. aeruginosa* K799/WT	21 ^d^	27	18	14	19
4	*P. aeruginosa* K799/61	24 ^d^	31	32	0	19

Results measured after the treatment with each compound (50 μL, 0.2 mM in 1:9 DMSO/MeOH) at 37 °C for 24 h. a = tested in the presence of 100 μM of 2,2′ bipyridine, an iron chelator, to simulate an iron-deficient environment. b = 5 μg/mL. c = 0.33 μg/mL in water. d = 1.66 μg/mL.

**Table 3:** Antibiotic activity of **S2a-W3** as measured by MIC (μM) values.

Entry	Strain	W3	S2a-W3
1	*A. baumannii* ATCC 17961	>128	0.125
2	*E. coli* ATCC 25922	2	8
3	*P. aeruginosa* ATCC 27853	>128	>128

**Table 4:** Antibiotic activity of **S2a-W9** and **S4-W9**, as measured by MIC (μM) values.

Entry	Strain	W9	S2a-W9	S4-W9
1	*A. baumannii* ATCC 17961	>100	0.4	0.2
2	*A. baumannii* ATCC 17978	-	-	0.8
3	*A. baumannii* BAA 1710	>100	0.8	-
4	*A. baumannii* BAA 1793	>100	0.8	-
5	*A. baumannii* BAA 1797	>100	0.8	-
6	*A. baumannii* BAA 1800	>100	0.8	-
7	*A. baumannii* ARC 3484	>100	0.4	3
8	*A. baumannii* ARC 3486	>100	0.4	3
9	*A. baumannii* ARC 5079	>100	0.8	12.5
10	*A. baumannii* ARC 5081	>100	0.4	12.5
11	*A. baumannii* ATCC 19606	-	0.8	-
12	*E. coli* DCO	>100	>100	>50
13	*P. aeruginosa* PAO1	>100	>100	-
14	*P. aeruginosa* KW799/WT	>50	-	>50
15	*P. aeruginosa* ARC 3502	>50	-	>50

**Table 5:** Antibacterial activity of **S3** and **S4** conjugates measured by MICs (µM).

Entry	Strain	W1		W2		S3a-W1		S3a-W2		S4-W1		S4-W2	
		+Fe	−Fe	+Fe	−Fe	+Fe	−Fe	+Fe	−Fe	+Fe	−Fe	+Fe	−Fe
1	*P. aeruginosa* PAO1	n.a.		n.a.		10	10	10	10	50	0.39	50	0.39
2	*E. coli* ATCC 25922	16.7	12.5	4.17	4.17	10	0.1	10	0.1	150	1.56	100	6.15
3	*E. coli* UTI89	10	10	10	10	1	0.1	10	0.1	-	-	-	-
4	*E. coli* CFT073	10	10	10	10	0.1	0.01	0.1	0.01	-	-	-	-
5	*E. coli* H9049	10	10	10	10	10	0.1	10	0.1	-	-	-	-
6	*E. coli* 35401	10	10	10	10	10	10	10	10	-	-	-	-
7	*E. coli* 43895	10	10	10	10	10	1	10	1	-	-	-	-
8	*E. coli* K-12	10	10	10	10	10	0.1	10	0.1	-	-	-	-
9	*E. coli* K-12 *fepA-*	10	10	10	10	10	10	10	10	-	-	-	-
10	*E. coli* K-12 *fepC-*	10	10	10	10	1	1	10	0.1	-	-	-	-
11	*E. coli* K-12 *fes-*	10	10	10	10	1	0.1	1	0.1	-	-	-	-
12	*P. aeruginosa* K799/WT	n.a.		n.a.		-	-	-	-	33	0.05	25	0.05
13	*P. aeruginosa* K799/61	0.52	0.78	0.46	0.39	-	-	-	-	12.5	0.067	12.5	0.083
14	*P. aeruginosa* Pa4	n.a.		n.a.		-	-	-	-	25	0.39	25	0.21
15	*P. aeruginosa* Pa6	n.a.		n.a.		-	-	-	-	n.a.	n.a.	n.a.	n.a.

n.a. = no activity.

**Table 6:** Antibiotic activity of **S3** conjugates measured by MICs (μM).

Entry	Strain	W6 (Cipro)	S3b-W6	S3c-W6 *	
				+Fe	−Fe
1	*E. coli* K-12	0.1	n.a.	n.a.	n.a.
2	*E. coli* B	0.1	n.a.	n.a.	n.a.
3	*E. coli* UTI89	0.1	n.a.	0.1	0.1
4	*E. coli* CFT073	0.1	n.a.	1	0.1
5	*E. coli* CFT073 *fepA-*	-	-	0.1	
6	*E. coli* CFT073 *iroN-*	-	-	0.1	
7	*E. coli* CFT073 *fepA- iroN-*	-	-	n.a.	
8	*E. coli* CFT073 *fepC-*	-	-	n.a.	
9	*E. coli* CFT073 *fepDG-*	-	-	n.a.	
10	*E. coli* CFT073 *fes-*	-	-	1	
11	*E. coli* CFT073 *iroD-*	-	-	n.a.	

n.a. = no activity up to 10 μM. * In tests against *E. coli* CFT073 mutants, **S3c-W6** was pre-loaded with 0.9 eq. of Fe(III).

**Table 7:** Antibiotic activity of catecholate conjugates **S5-S13** measured by MIC values (mg/L).

Entry	Strain	W1	W5	S5-W1	S6-W1	S7-W1	S8-W1	S9-W1	S10-W4	S11-W4	S12-W1	S13-W1	S13-W2	S13-W5
1	*E. coli* ATCC 25922	6.26	12.5	0.78	0.4	0.2	0.4	0.1	n.a.	100	50	6.25	3.12	1.56
2	*P. aeruginosa* ATCC 27853	n.a.	100	0.4	0.4	0.78	0.4	0.2	3.12	25	6.25	0.78	6.25	50
3	*P. aeruginosa* SG 137	n.a.	100	<0.05	<0.05	0.05	<0.05	0.01	<0.05	12.5	0.4	0.2	0.78	50

n.a. = no activity up to 100 mg/L.

**Table 8:** Antibiotic activity of **S14** conjugates measured by MIC values (μM).

Entry	Strain	W10	S14a-W10	S14b-W10
1	*A. baumannii* ATCC 17961	n.a.	n.a.	0.8
2	*A. baumannii* ATCC BAA 1793	n.a.	n.a.	0.8–0.16
3	*A. baumannii* ATCC BAA 1797	n.a.	n.a.	6.25
4	*A. baumannii* ATCC BAA 1800	n.a.	n.a.	0.8
5	*E. coli* DC0	n.a.	n.a.	<0.025
6	*P. aeruginosa* KW799/WT	n.a.	n.a.	0.2–0.4

n.a. = no activity up to 50 μM.

**Table 9:** MIC_50_ (mM)—antibacterial activity of effective pyochelin–drug conjugates.

Entry	Strain	W6		W7		W8		S15f-W6		S15f-W7	
		+Fe	−Fe	+Fe	−Fe	+Fe	−Fe	+Fe	−Fe	+Fe	−Fe
1	*P. aeruginosa* PAO1	0.060	0.040	0.200	0.110	0.350	0.200	0.700	0.600	1.000	n.a.
2	*P. aeruginosa* PAD07 (*pvd*- *pch*-)	0.045	0.060	0.190	0.200	0.360	0.350	0.600	0.700	1.000	1.000
3	*P. aeruginosa* PAD14 (*tonB*-)	0.040	0.035	0.120	0.120	0.210	0.180	0.200	0.170	0.550	0.450

## Data Availability

No new data were created for this manuscript.
